# Sequence Alignment, Mutual Information, and Dissimilarity Measures for Constructing Phylogenies

**DOI:** 10.1371/journal.pone.0014373

**Published:** 2011-01-04

**Authors:** Orion Penner, Peter Grassberger, Maya Paczuski

**Affiliations:** 1 Complexity Science Group, Department of Physics and Astronomy, University of Calgary, Calgary, Alberta, Canada; 2 Department of Biological Sciences, Institute for Biocomplexity and Informatics, University of Calgary, Calgary, Alberta, Canada; University of Stellenbosch, South Africa

## Abstract

**Background:**

Existing sequence alignment algorithms use heuristic scoring schemes based on biological expertise, which cannot be used as objective distance metrics. As a result one relies on crude measures, like the p- or log-det distances, or makes explicit, and often too simplistic, *a priori* assumptions about sequence evolution. Information theory provides an alternative, in the form of mutual information (MI). MI is, in principle, an objective and model independent similarity measure, but it is not widely used in this context and no algorithm for extracting MI from a given alignment (without assuming an evolutionary model) is known. MI can be estimated without alignments, by concatenating and zipping sequences, but so far this has only produced estimates with uncontrolled errors, despite the fact that the *normalized compression distance* based on it has shown promising results.

**Results:**

We describe a simple approach to get robust estimates of MI from *global pairwise* alignments. Our main result uses algorithmic (Kolmogorov) information theory, but we show that similar results can also be obtained from Shannon theory. For animal mitochondrial DNA our approach uses the alignments made by popular global alignment algorithms to produce MI estimates that are strikingly close to estimates obtained from the alignment free methods mentioned above. We point out that, due to the fact that it is not additive, normalized compression distance is not an optimal metric for phylogenetics but we propose a simple modification that overcomes the issue of additivity. We test several versions of our MI based distance measures on a large number of randomly chosen quartets and demonstrate that they all perform better than traditional measures like the Kimura or log-det (resp. paralinear) distances.

**Conclusions:**

Several versions of MI based distances outperform conventional distances in distance-based phylogeny. Even a simplified version based on single letter Shannon entropies, which can be easily incorporated in existing software packages, gave superior results throughout the entire animal kingdom. But we see the main virtue of our approach in a more general way. For example, it can also help to judge the relative merits of different alignment algorithms, by estimating the significance of specific alignments. It strongly suggests that information theory concepts can be exploited further in sequence analysis.

## Introduction

Sequence alignment achieves many purposes and comes in several different varieties [1]: Local versus global (and even “glocal”: [2]), pairwise versus multiple, and DNA/RNA versus proteins. Rather than listing all applications, we cite just two numbers: According to Google Scholar the two original papers on the BLAST algorithm for local alignment by [3] and on one of its improvements [4] have been cited more than 30,000 times each, and the number of daily file uploads to the NCBI server providing BLAST is 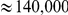 [5]. A partial list of alignment tools in the public domain can be found in http://pbil.univ-lyon1.fr/alignment.html.

In *global* alignment, which we focus on here, two sequences of comparable length are placed one below the other. The algorithm inserts blanks in each of the sequences such that the number of positions at which the two sequences agree is maximized. More precisely, a *scoring scheme* is used. Each position at which the two sequences agree is rewarded by a positive score, while each disagreement (“mutation”) and each insertion of a blank (“gap”) is punished by a negative one. The best alignment is that with the highest total score. In *local* alignment, one aligns only subsequences against each other and looks for the highest scores between any pairs of subsequences. Regions that cannot be well-aligned are simply ignored. Existing algorithms use either heuristic scoring schemes or scores derived from explicit probabilistic models [6].

Similarities between DNA sequences, e.g. for distance-based phylogenetic tree construction, are typically not based on alignment scores. Instead they use explicit evolutionary assumptions (e.g. the Kimura two-parameter model [7]) or are simply obtained by counting the number of nucleotide substitutions (like the p-distance or the Poisson corrected p-distance [7]). An important property of a similarity measure, from the point of view of phylogeny, is that distances should grow linearly with evolution time. This results in a measure satisfying the so-called *four point condition* [8], which in turn makes the measure useful for *neighbor joining*, the most popular distance based algorithm for inferring phylogenetic trees [9]. The most important metrics developed from this view point are the closely related *paralinear* [10] and *log-det* [11] distances. In this paper we refer to both as “log-det”, for simplicity's sake.

In the above mentioned distances, distinct rates of different substitution types are either taken into account using a model, or are not taken into account at all. This fact stands in stark contrast with *mutual information* (MI), which takes the amount of information shared between two objects as a measure of their similarity [12]. For instance, more frequent substitutions can be encoded more efficiently, and should thus be a weaker indicator for dissimilarity than rare, and thus “surprising”, substitutions. The crucial point to note is that the frequency of substitutions and indels and their correlations can be counted directly from the alignment, and no model is required. As a consequence, MI is, in principle, a model-free, universal, and objective similarity measure, in stark contrast to all metrics discussed above.

Indeed, there are two variants of information theory: The more traditional Shannon theory, based on a probabilistic interpretation of the sequences, and the less well known Kolmogorov (or *algorithmic*) “complexity” theory [12]. In this paper we use Kolmogorov information as our main vehicle, but we also show that Shannon theory gives comparable results.

Roughly, in algorithmic information theory the *complexity* 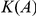 of a sequence 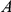 is the minimal amount of information (measured in bits) needed to specify 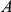 uniquely, on a given computer, with a given operating system. Numerical results depend on the latter, but this dependence will, in general, be weak and is ignored in this paper. For two sequences 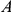 and 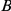, the conditional complexity (or conditional information) 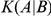 is the information needed to specify 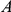, if 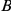 is already known (i.e., either it or its specification was already input before). If 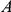 and 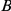 are similar, this information might consist of a short list of changes needed to go from 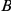 to 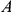, and 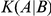 is small. If, on the other hand, 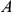 and 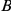 have nothing in common, then knowing 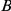 is useless and 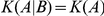. Finally, the *mutual information* (MI) is defined as the difference 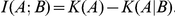 It is the amount of information which is common to 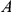 and 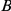, and is also equal to the amount of information in 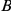 which is useful for describing 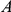, and *vice versa*. Indeed, it can be shown that, up to correction terms that become negligible for long sequences (see [12]): (a) 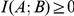; (b) 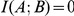 if and only if 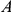 and 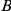 are completely independent; (c) 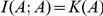; and (d) 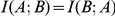. Moreover, the probability (in the Bayesian sense) that 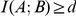 is smaller than 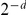, if 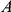 and 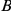 are independent (see Theorem 2 of [13]). Hence, the similarity is significant and not by chance when 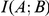 is large.

The fundamental difference between Shannon theory and Algorithmic Information theory is that Shannon theory makes no attempt to quantify the minimum information required to specify a any particular sequence. Instead Shannon theory assumes that a sequence can be treated as though it was generated as a “typical” case of a probabilistic process. As a result of this assumption, Shannon information has no dependence on hardware. However, the main drawback is that it cannot, strictly speaking, deal with individual sequences and it needs an assumption on the probability distribution. Any numerical result obtained from individual sequences implies the assumption that the specific sequences are ‘typical’ of the underlying probabilistic process. As a result it involves statistical inference, even if the result does not strongly depend on this inference. In particular, the assumption of independence of letters in a sequence (used also below) will lead to over-estimation of Shannon entropy, and thus implies no risk of overfitting.

The fact that alignment and information theory are closely related has been realized repeatedly. However, most work in this direction has focused on aligning images rather than sequences [14]. Conceptually, these two problems are closely related, but technically, they are not. The effects of sequence randomness on the significance of alignments has also been studied in [15]. Finally, attempts to extend the notion of *edit distance* [1] to more general editing operations have been made. In this case the similarity of two sequences is quantified by the complexity of the edit string, see [16]. Indeed, the aims of [16] are similar to ours, but their approach differs in several key respects and leads to markedly different results.

## Methods

### Translation Strings

**Figure 1:**
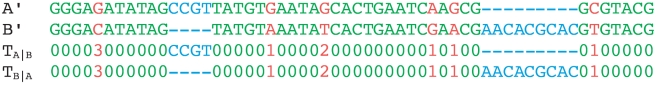
Example of an alignment and of the two translation strings 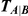 and 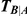. Colors indicate sites with mutations (red), gaps (blue), and conservation (green).


At the heart of our approach is the concept of a *translation string*. The translation string 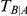 contains the minimal information necessary to recover the sequence 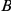 from another sequence 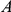. Similarly, 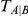 contains the information needed to obtain 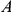 from 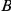. Here we focus on DNA sequences, consisting of the letters A,C,G and T, and corresponding to complete mitochondrial genomes. But the approach is more general and can be applied to protein sequences without further effort. We refer to the 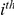 element of sequence 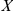 as 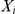, and denote the length of 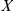 as 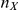. Any global alignment algorithm, when applied to 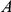 and 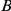, outputs a pair of sequences 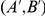 of equal length 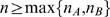. The sequences 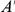 and 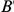 are obtained from 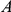 and 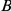 by inserting hyphens (“gaps”) such that the total score is maximized. The strings 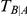 and 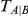 also have length 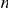, and are composed from an alphabet of nine characters. For each 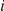, the letter 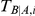 is a function of 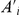 and 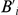 only. An example of this process is found in Figure 1; the rules to create 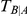 are as follows:

- if 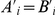, then 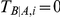;
- if 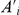 is a hyphen (gap), then 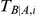 has to specify explicitly what is in 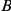; hence 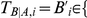
A,C,G,T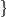;
- if 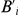 is a hyphen (gap), then 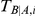 has to indicate that *something* is deleted from 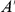, but there is no need to specify what. Hence 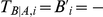;
- if 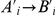 is a *transition*, i.e. a substitution A
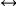
G or C
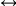
T, then 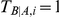;
- if 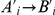 is a *transversion* A
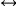
C or T
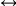
G, then 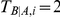;
- if 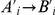 is a transversion A
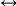
T or G
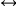
C, then 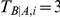.

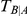
 is defined such that 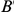 (and thus also 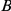) is obtained uniquely from 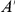. But 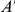 can be obtained from 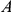 using 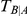. Thus 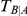 does exactly what it is intended to do: it allows one to recover 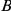 from 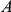. It does not, however, allow one to recover 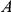 from 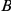. Due to the second and third bullet points above, 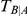 is not the same as 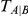. This distinguishes our approach from typical edit string methods.

### Algorithmic Information Theory: Mutual Information

An estimate of the conditional complexity 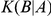 is obtained by compressing 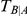 using any general purpose compression algorithm such as zip, gzip, bzip2, etc. In the results shown here we use lpaq1 [17] (see also this reference for a survey of public domain lossless compression algorithms). Denoting by 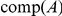 the compressed version of 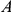 and by 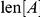 the length of 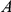 in bits, gives an exact upper bound
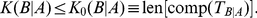
(1)If there were no correlations between sequence 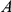 and the translation string, 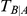, this would also be the best possible upper bound. However, in general we must expect that such correlations exist, although we find them to be weak (see the second figure in Material S1). Thus Eq. (1) is still a good estimate, but the best one is obtained by compressing conditionally on 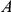,
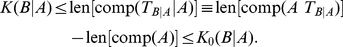
(2)More precisely, one can show that 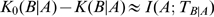.

In order to obtain an estimate of MI, we have to subtract 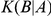 from 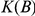, which is also estimated via compression. Unlike 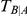, 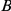 is a DNA string. Since general purpose compression algorithms are known to be inferior for DNA [18], [19] we could use an efficient DNA compressor like, e.g., ‘GeneCompress” or “XM” [18], [19] (as we shall do in Eq. (4) below). To avoid any question of consistency, we shall not do this. Instead, the compression is carried out using a general purpose compression algorithm, to get
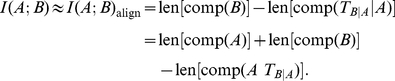
(3)


This is to be compared to the general definition of algorithmic MI, based entirely on concatenation and compression [12], [20] without using any alignment. This estimate is obtained by comparing the size of the compressed concatenation 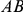 to the sum of the sizes of the compressed individual files,
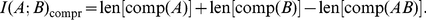
(4)


At first sight it might seem paradoxical that 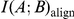 can even be positive. Not only does 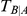 involve a larger alphabet than 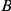, but, in general, it is also a longer string. Thus one could expect that 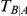 would not typically compress to a shorter size than 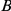. The reason why this first impression is wrong is clear from Figure 1: If 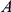 and 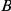 are similar, then 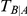 consists mostly of zeroes and compresses readily. In practical alignment schemes, the scores for mismatches are carefully chosen such that more frequent substitutions are punished less than unlikely substitutions. In contrast, coding each mismatch simply by a letter in 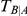 seems to ignore this issue. However, more frequent mismatches will give letters occurring with higher frequency, and general purpose compression algorithms utilize frequency differences to achieve higher compression.

Conceptually our approach is similar to encoding generalized edit strings in [16]. However, there are several pivotal differences between that work and ours. First, the authors in [16] did not compress their edit strings and as a result the conclusions they were able to draw from a quantitative analysis were much weaker than ours. Second, our approach utilizes an alignment algorithm to achieve an efficient encoding of 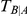. In addition to producing a better estimate of 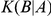, this allows us to make quantitative evaluations of the alignment algorithm itself. An additional difference between our approach and the traditional edit methods used in approximate string matching [21] is that our *translation strings* do not give both translations 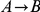 and 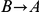 from the same string. This asymmetry is crucial to establish the relations to conditional and mutual information.

For long strings, 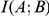 should be symmetric in its arguments. In general, the estimates satisfy 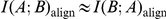 (see the third figure in Material S1). Indeed, the translation strings 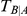 and 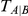 can differ substantially, resulting in different estimates for 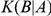 and 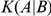 via Eq. (2). This difference is mostly canceled by differences between 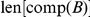 and 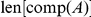. Take, for instance, the case where 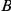 is much shorter than 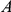. Then 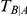 consists mostly of hyphens and is highly compressible. On the other hand, 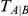 is similar to 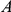, since most letters have to be inserted when translating 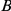 to 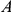. Thus both 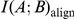 and 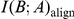 are small compared to 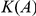, but for different reasons. Further details are given in Material S1.

### Shannon Theory

Compared to algorithmic information, Shannon theory is the more widely known version of information theory [12]. The basic concept of Shannon theory is that of a *block* or *word* probability 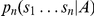. It gives the probability that the ‘word’ 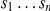 of 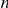 consecutive letters (such as A,C,G or T for DNA) appears at any random position in the string 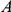. Here we assume stationarity, but we do not assume absence of correlations. The entropy (analogous to the complexity in algorithmic information theory) of a string comprised of letters from an alphabet 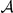 is defined as 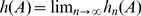 with
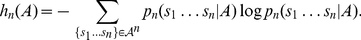
(5)From this, MI is defined as in algorithmic theory: 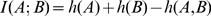 [12]. If entropy is measured in bits, then the logarithm is to base 2. In practice, the limit 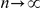 is rarely feasible, and one usually approximates 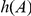 by the single-letter entropy
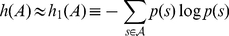
(6)or, at most, by the pair approximation based on the probabilities for words of length two.

Eq. (6) is valid under the assumption that correlations between consecutive letters in the string can be neglected. Similarly, 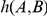 for two sequences of equal length is estimated by assuming that consecutive letter pairs 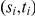 with 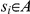 and 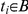 are independent. If we make this assumption, there are still two ways to estimate the MI of two strings. In the first we use the fact that 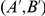 carries the same information as 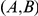 to employ a five-letter alphabet 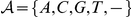. This has the drawback that indels are usually correlated. In the second we thus neglect all indels and reduce the alphabet to 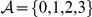. In the following we shall mostly use the latter to compare with other pairwise distance metrics, but we stress that we do this only for simplicity and convenience (and since it is sufficient to make our point). However, the more interesting MI estimate remains the one obtained from algorithmic theory, due to the fact it takes into account both indels and all possible correlations within each string and between them.

### Distances, Trees and Quartets

The value of the MI itself is useful for many purposes: Estimating similarities between different pairs (and thus of finding closest neighbors of a given sequence in a large data set); comparing the qualities of alignments obtained by different algorithms; or assessing the significance of an alignment (i.e., verifying that it is better than an alignment between two unrelated sequences). But in the case of phylogeny, one wants more. Ideally, one wants an *additive metric distance*, i.e. a non-negative symmetric pairwise function 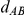 for which 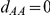 and which satisfies both the triangle inequality
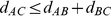
(7)for any triple, and the *four-point condition* [22]

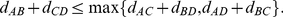
(8)for any quartet. The latter is a necessary and sufficient condition for all pairwise distances between 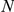 sequences to be representable as distance sums over links in a tree [8] with the 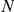 sequences represented by the leaves. Thus distances satisfying Eq. (8) are also called ‘tree metrics’.

Several potential metrics can be derived from MI [20], [23], [24]. According to [20], [24], the preferred one is the *normalized compression distance*

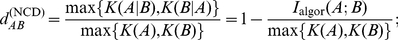
(9)where 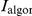 can be either 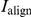 or 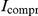, depending on the way it is estimated. For Shannon theory we can use the same construct with 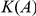 replaced by 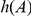 [25]. Since it would be confusing to use the word “compression” for this metric, we have to use another name. We call it the *normalized Shannon distance*

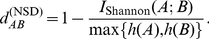
(10)


Although 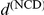 has been used to produce meaningful phylogenetic trees [20], [23]–[25], it has one important drawback for phylogenetic applications: It is not additive. Indeed, for two completely unrelated sequences (corresponding to infinite evolutionary distance), both 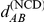 and 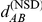 do not go to infinity, but rather to 1. They are not linear but convex functions of evolutionary distance. Such metrics are well known to lead to *long branch attraction* (or the ‘Felsenstein phenomenon’ [26]).

If evolution is assumed to be a Markov process, then the data processing inequality [12] guarantees that MI decreases with evolutionary distance. A natural assumption – following from the dominance of a single maximal eigenvalue of the Markov matrix – is that it decreases exponentially to zero. In this case the *log-MI* “distance”
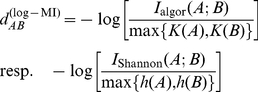
(11)would increase linearly with evolution and would be thus additive. Unfortunately, 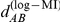 is not a proper metric, as it does not even satisfy the triangle inequality. This can be seen from the following example: Take three sequences over an alphabet of four letters (like DNA) where each letter is represented by two bits (purine/pyrimidine, double/triple hydrogen bonds). Sequence 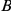 is random, sequence 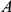 is obtained from 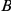 by replacing randomly the first bit but conserving the second, and 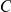 is obtained by replacing the second but conserving the first. Then 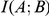 and 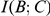 are non-zero, while 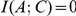. At the same time, all single sequence complexities (and entropies) are the same, thus 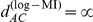 while 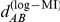 and 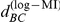 are finite, clearly violating Eq. (7).

Fortunately, real evolution is most likely not as extreme as this counter example, and the triangle inequality is not really required for distance based phylogeny. In particular, the relationship between trees and metric additivity is not restricted to metrics satisfying the triangle inequality, as seen from the proof in [8]. Also, the neighbor joining algorithm [9] does not require the triangle inequality. Thus we claim that 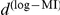 is an *a priori* better distance measure for phylogeny than 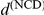 or 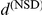, although a final evaluation can only be made through detailed tests on real biological sequences.

Such tests are presented in the results section, with the log-det (or, more precisely, the paralinear) distance [10], [11] and two distances based on Kimura's model [7] (see the supplementary information) as other competitors. In the latter, one assumes different rates 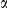 for transitions (A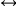 G, C
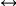
T) and 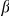 for transversions (all others).

Assume that for two aligned sequences, 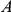 and 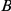, one first eliminates all positions with indels. Thus, at each site one sees one of the 16 possibilities 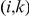 with 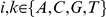. Denote the measured frequencies for these possibilities 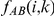. The single-sequence (‘marginal’) frequencies are 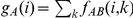 and 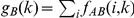. We introduce matrices 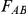 with matrix elements 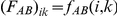, 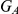 with 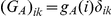, and 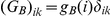 (here, 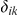 is the Kronecker delta, i.e. 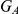 and 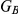 are diagonal matrices). The log-det distance is then defined as
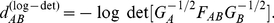
(12)In [10], this is called paralinear distance; in [11] the name log-det is used either for this or for simplified versions where the matrices 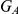 and 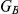 are omitted. This difference is irrelevant for additivity and for use in the neighbor joining algorithm. It can be shown that 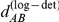 is additive under rather general evolutionary models, although not when evolutionary speed is site dependent.

Before moving on, we should point out that the data required to compute the log-det distance are *precisely* the same as those needed to compute the two MI-based distances 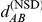 and 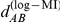, provided one uses for the latter the single-letter Shannon formulas with indels deleted. In that case,
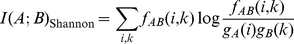
(13)and
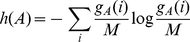
(14)where 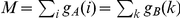, and is the number of sites in the alignment. This is the main reason we will later compare these three distances in detail.

### Tools

We utilized the MAVID [27] and Kalign [28] global sequence alignment programs available for download at [29] and [30]. We also experimented with STRETCHER [31], lagan [32] and CLUSTALW 2 [33], and observed similar results. We made no effort to optimize the scoring parameters of the algorithms and only used the default values.

To evaluate 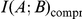 we utilized the *expert model* (XM) DNA compression algorithm [19]. To evaluate 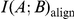 we used lpaq1 [17]. Using lpaq1 was not crucial, with the standard LINUX tools gzip and bzip2 producing similar results. For DNA we also explored GenCompress [23] and bzip2. Both showed markedly inferior results to XM (see supplementary information), although their ability to compress single sequences is not so much inferior to XM [19]. Presumably this is due to the fact that XM is more efficient in finding and exploiting approximate repeats, which is crucial in compressing concatenated strings.

The complete mtDNA sequences used in our analysis were downloaded from [34]. We paid special attention to eliminate incomplete sequences and sequences with too many wild cards. We also took care to circularly shift the sequences (mtDNA forms in most cases a closed ring) in order to improve the alignments. We used different subsets of sequences for different plots. In a few cases we also flipped the strands, if this led to much better alignments. Overall, we used nearly 1800 sequences.

## Results

### Alignment based mutual informations versus compression based mutual informations

**Figure 2:**
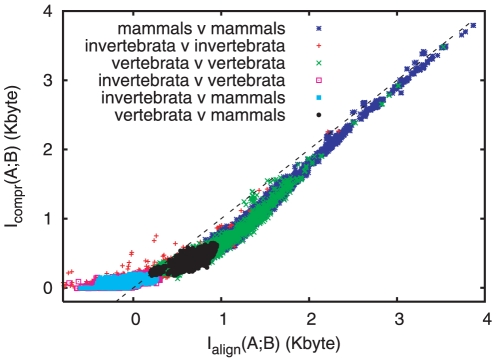
Scatter plot of MI estimates for complete mitochondrial DNA between pairs of species: 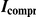 using XM [19] vs. 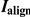 using MAVID [27] followed by compression with lpaq1. Note that the two estimates generally agree and fall on the diagonal, while in some cases one method does better than the other as explained in the text. Here and in Figure 3 “vertebrata” means non-mammalian vertebrata. This plot contains roughly 36,000 pairs, about 16,000 of which contain two mammals, the other 20,000 covering equally the other combinations. 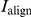 is the average between the values obtained from 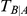 and 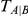.

Our first results concern the agreement between the two estimates 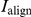 and 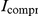. In Figure 2 we compare estimates 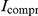 obtained with XM to estimates 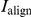 obtained with the MAVID alignment tool [29] and with subsequent compression using lpaq1. It is well known that DNA and amino acid sequences are hard to compress [18], [19], thus one might expect that 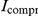 depends strongly on the compression algorithm used. This is indeed the case, as seen from the first figure in Material S1, where we compare values of 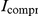 obtained with three different compression algorithms: The general purpose compressor lpaq1 [17] and the two special DNA compressors GeneCompress [18] and XM [19]. From this figure it is clear that XM is far better the other two. Note that it is very likely that an imperfect compression algorithm underestimates rather than overestimates MI – although we do not know a rigorous theorem to this effect.

In view of this, it is not obvious that the estimates produced by XM are realistic either. It is thus highly significant that the two estimates shown in Figure 2 are approximately equal, despite the fact that alignment algorithms and compression algorithms follow drastically different routes. The slight downward shift from the diagonal, particularly visible for large MI pairs, is due to an off-set of 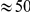 bytes in the XM algorithm. Points above the diagonal indicate that concatenation and compression – using the XM algorithm – produce a better estimate of MI, while points below indicate that MAVID alignment followed by compression of its translation string produced a better estimate. The invertebrate-invertebrate pairs far above the diagonal in Figure 2 correspond to pairs of species where the individual genes are similar, but their ordering is changed (this refers in particular to all pairs with 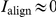 and 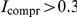 Kbyte). In that case a compression algorithm is superior to a global alignment algorithm, since it is not affected by shuffling open reading frames (ORFs). Most negative estimates for MI seen in Figure 2 represent cases where shuffling the ORFs prevented reasonable global alignments. Particularly interesting are pairs of mammals with 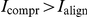. We checked that *all* of them involve a subspecies of sikka deer (Cervus nippon taiouanus, GenBank accession number DQ985076), in which a single gene (NADH6) is supposedly on the opposite strand compared to all other mammals.

Agreement between 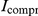 and 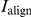 could have been improved presumably in many cases by masking part of the genome, but we have not tried this. In any case, the occasional disagreements are of particular interest, since they indicate where one of the two approaches encountered particular difficulty. Generally speaking Figure 2 suggests that DNA compression can still be improved slightly, as seen from pairs with 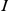 between 1 and 2 Kbyte (corresponding roughly to species in different families but the same orders). On the other hand, purely compression based MI estimates give non-trivial (at least positive) results even across different classes.

### Comparison between different alignment algorithms

**Figure 3:**
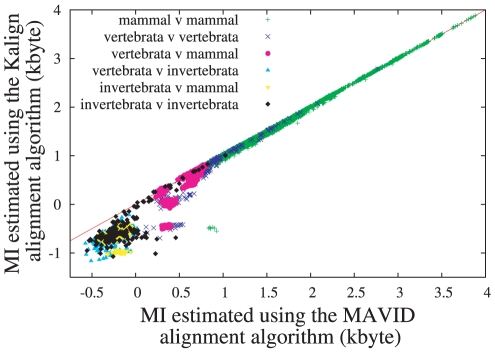
Scatter plot comparing alignment based MI estimates: Kalign [28] vs. MAVID [27]. The number of pairs here is about half of that shown in Figure 2. Points on the diagonal indicate agreement between the two estimates. These data were generated using the default scoring parameters. Therefore, the plot represents a proof of principle for using MI to evaluate alignments rather than a definitive statement about the quality of the two alignment algorithms shown.

MI estimates obtained using other global alignment algorithms are similar to those obtained with MAVID; an example is shown in Figure 3. In this figure we see that MAVID produced slightly, but systematically better alignments. However, because neither algorithm's scoring scheme was optimized, we do not consider this figure to indicate which of the two alignment algorithms is better. Rather, it represents a proof of principle that our method can be used to identify strengths and weakness of different alignment algorithms and evaluate objectively the sequence similarity in any given alignment.

### Correlations within single translation strings: Shannon informations

**Figure 4:**
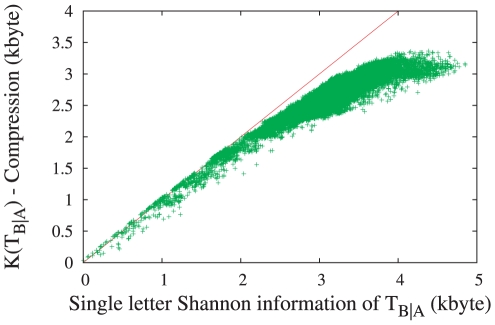
Scatter plot comparing 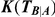 estimated using compression, to the single letter Shannon information 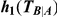. The diagonal, 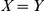, is a guide for the eye. Points falling below the diagonal indicate cases where 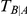 is not independent and identically distributed, and some letters show strong correlations. The fact that 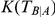 is slightly larger than 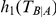 for low entropy translation strings corresponds to the initiation cost for lpaq1 compression, which is 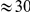 byte independently of the sequence. The plot shows 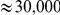 pairs taken from all over the animal kingdom.

In Figure 4 we show compression based conditional complexity estimates for animal mtDNA translation strings plotted against the corresponding single letter Shannon entropies 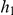. In the latter, we have not eliminated indels, i.e. they are based on the nine letter alphabet 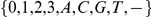. Thus the difference between 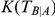 and 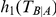 is entirely based on correlations, detected by the compression algorithm (in this case lpaq1).

As 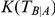 goes to zero, the two estimates agree, up to a small initialization cost for lpaq1 of 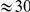 bytes. The estimates agree because the translation string is mostly composed of zeros, with the few substitutions being far apart and weakly correlated. For increasing 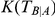, however, the compression algorithm often gives significantly lower estimates, thus indicating strong correlations within the translation string. More detailed analysis of pairwise correlations (unpublished) suggests that these are mostly correlations between letters 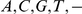 (i.e., inserts and gaps rather than substitutions). The fact that indels occur strongly correlated is well known [1], and is also assumed in most alignment scoring schemes.

Therefore, if the information encoded in gaps is to be taken into account, it is necessary to go beyond the single letter approximation when estimating realistic and absolute sequence similarities. Furthermore, taking into account only pairwise letter correlations would not be sufficient either. This, of course, is not completely new, and the most common way to deal with this problem is to simply ignore indels [7]. Indeed there seems to exist a wide spread opinion that indels are not very informative and useful. Whether this is true or whether it just reflects an inability to deal with this information efficiently is an open question. In any case, the most straightforward way to deal with it would be based on algorithms using data compression.

### Comparison with p-distances: The effect of indels

A very simple but popular distance measure between sequences (both DNA and amino acids) is p-distance. It is defined by first removing all indel positions and then counting the number of positions where the two sequences disagree [7],
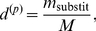
(15)where 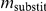 is the number of observed substitutions and 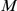 is the total number of (non-indel) sites. Since this quantity saturates with increasing evolutionary distance, a slightly more sophisticated version is the Poisson corrected (PC) p-distance [7], 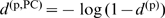. We note that neither 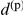 nor 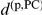 take into account the type of substitutions, any information contained in indels, or any information contained in internal correlations within the translation strings.

**Figure 5:**
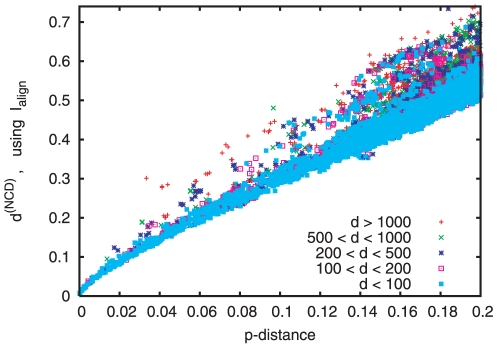
Scatter plot comparing p-distances 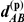 to normalized compression distances 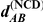 obtained from 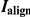. The figure is based on 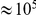 mtDNA pairs, selected according to criteria discussed in the main text. Different symbols correspond to different length differences 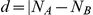, where 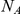 and 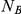 are the original (non-aligned) sequence lengths.

Our main interest here is to see which of these three neglected aspects (type of substitution, indels, correlations) has the biggest effect. In Figure 5 we show a scatter plot of the normalized compression distance 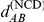, estimated via 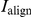, against 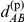, for 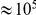 pairs 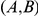 taken from all over the animal kingdom. In order to avoid meaningless alignments, we took in each pair only members in the same (sub-) phylum (hexapoda, mollusca, crustacea, chelicerata, cnidaria, porifera, platyhelminthes, echinodermata) or in the same (super-) class (mammals, sauropsida, amphibia, actinopterygii). We also eliminated pairs with 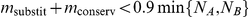, as we would have otherwise too many biologically meaningless alignments. Here, 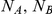 are the sequence lengths; this criterion guarantees that there are not too many insertions into the longer sequence, and not too many deletions from the shorter. We found that there is a roughly monotonic relationship between 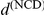 and 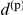, with occasional, strong, deviations. By far the strongest factor leading to these deviations is the difference in length of the paired raw (i.e. unaligned) sequences. Nearly all gross outliers in Figure 5 correspond to pairs in which one member has a very long mitochondrial genome, leading to a large number of indels.

As we had pointed out in the previous subsection, it is widely believed that indels are not very informative. We plan to check this more carefully in a future publication, using a methodology based on a large number of quartets for sequences similar to the one described in the next section.

### Comparison with log-det distances: The effect of substitution types

Finally, we want to compare our distance metrics 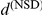 and 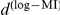 to the log-det distance 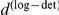 given in Eq. (12). In order to simplify the discussion and to use exactly the same input for all three metrics, we use the same alignment algorithm (MAVID) for each pair and delete all indels. As mentioned above, 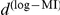 does not, in general, satisfy the triangle inequality. But this does not preclude it from being satisfied in all “typical cases”. To test this we first check whether the triangle inequality is actually violated or not in 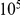 randomly chosen triplets, drawn from the entire animal kingdom, with the same selection criteria as in the previous subsection. Note that due to the omission of indels none of these “distances” actually have to satisfy the triangle inequality. Indeed, we found 11 violations for the log-det distance, and none for either of the MI-based distances.

**Figure 6:**
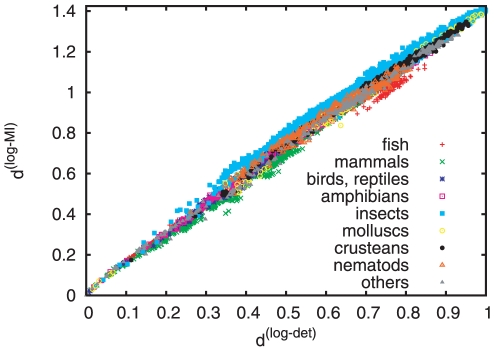
Scatter plot comparing 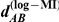 (based on single letter Shannon entropies) to 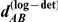, for 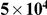 randomly chosen pairs of species.

Next we tried to check whether 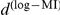 is at least approximately additive. Since we do not have the true evolutionary distances, we take 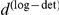 as a proxy. In Figure 6 we plot 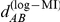 against 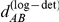 for 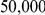 random pairs. We see that:

- Roughly, the dependence is linear. Thus, to the extent that 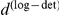 is linear, 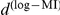 is too. Thus it should not be affected by long branch attraction. This is in contrast to 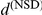 which – when plotted against 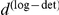 – is strongly non-linear (data not shown).
- On a finer scale, one sees several deviations. The most conspicuous, perhaps, is that insects (hexapods) are systematically above the main curve. This is due to the strong compositional bias in most insects, where C/G is underrepresented compared to A/T. This reduces the entropies of individual sequences. At the same time, however, substitution rates involving C and G are not as suppressed. As a consequence, the ratio 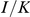 is enhanced compared to other phyla, and 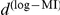 is increased. This is a desirable effect. It is well known [35] that similar compositional bias can make two sequences look more closely related, even if they are not closely related evolutionarily. While this applies fully to 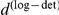, the effect is at least smaller for 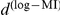.
- For intermediate distances (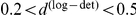), many mammals are below the main line. In particular, consider the two pairs well below it at 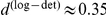. Both involve the spectacled bear (Tremarctos ornatus) and another Ursinae species. For whatever reasons, these two translation strings contain an unusually large ratio of transitions to transversions that would otherwise only be typical for much more closely related species. This reduces the information content when compared to unbiased substitutions with the same total frequencies. At the same time, the individual sequences are not very strongly biased. Thus 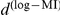 is reduced, but 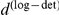 is not – since it is only weakly dependent on the detailed substitution rates. Again we claim that this favors 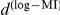 over 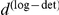.

A clear decision whether this is indeed true can only be made by detailed comparison of phylogenies predicted on the basis of these metrics with the true phylogenies. Since the latter are of course unknown, we take inferences made in the literature as proxies. Our detailed strategy is the following:

1. We first choose 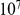 random quadruples from all over the animal kingdom. We use the same taxonometric restrictions, to avoid too many pairs which cannot be meaningfully aligned. Thus each quadruple (or “quartet”) contains only species from the same (sub-)phylum or the same (super-)class, respectively. We also used the same cut on the number of indels, in order to eliminate false alignments.
2. For each quartet, we find the topologies suggested by each of the three metrics, and count the number of cases where two metrics disagree. This gave 185543 quartets (1.9%) where 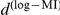 and 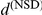 disagree, 429386 quartets (4.3%) where 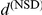 and 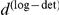 disagree, and 380487 quartets (3.8%) where 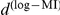 and 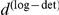 disagree.
3. For each quartet we compute a significance 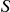 with which the suggested topology is actually preferred. This significance is explained in detail in the supplementary information. It involves both the amount by which the four-point condition is violated, and the relative length of the central edge, if the data are approximated by an additive tree. For each pair of metrics we then pick the quartets for which the metrics disagree most significantly (as measured by the sum of the two significances). Actually, we do not strictly choose the worst disagreements, as they would cluster within a few taxa and we want our results to represent as much of the entire animal kingdom as possible. As such, we take relatively more quartets in taxa which are underrepresented in GenBank, and we reject quartets (not entirely systematically) if three of the four species had already appeared in many selected quartets.
4. In this way we selected 129 “worst” disagreements between 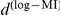 and 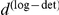, and 129 “worst” disagreements between 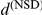 and 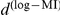. For reasons that will become clear later, we did not select worst disagreements between 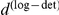 and 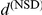, except for a few cases. For each of these worst cases we searched the literature and established the “correct” topology. Details are again given in the tables found in the Material S1.

The final results of this are summarized in Table 1. They clearly indicate that the log-MI metric is vastly superior the log-det distance, in spite of the latter's superior theoretical foundations. This is at odds with the fact that the log-MI metric is not a proper distance, and does not, in any reasonable model, satisfy the four-point condition (Eq. (8)). The reason obviously is that 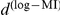 takes into account, in an optimal model-independent manner, compositional details that 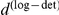 does not. The comparison between 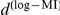 and 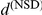 is much less clear. One might have expected that the strong non-additivity of 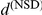 makes it unsuitable for this sort of phylogenetic application. But this is not so clear; 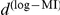 is only marginally better. This seems surprising, but a possible reason for it will be given in the discussion.

**Table 1:** Number of quartets for which each pairwise metric produces a topology that agrees better with that found in the literature.

	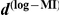 versus 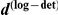	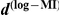 versus 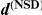
first agrees	106	57
second agrees	17	42
neither	3	24
undecided	3	6

The quartets examined are among those for which the disagreement between the two metrics is quantitatively the worst. We note that, when compared to 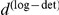, 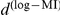 produces a topology that agrees with literature much more often. “Neither” indicates the case where neither metric produces a topology that agrees with the current literature. “Undecided” indicates that it is not possible to establish a ‘correct’ topology on the basis of current literature.

Before moving on we highlight a few notable observations about our quartet analysis. Previously, we pointed out that the spectacled bear (T. ornatus) is anomalous either in 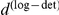 or in 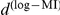. Indeed, it appears twice in the first table of the supplementary, and both times 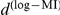 gives the correct grouping. A similar anomaly is seen in Figure 6 for fish (actinopterygii) at 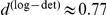. Most of these correspond to Albula glossodonta (GenBank AP002973) paired with other fish. The second table of Material S1 shows that for most of these pairs the log-MI distance gives a better estimate.

We find that discrepancies between 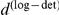 and 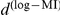 are very unevenly distributed over the taxa. While we found no disagreements in the chaetognatha, there are a large number in the nematods, most favoring 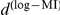. Indeed, it seems that the nematod phylogenetic tree constructed using 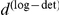 would be *systematically* different from the tree constructed using 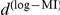 and other analyses.

It is well known [10], [11] that the log-det distance is additive only when the evolutionary rate is constant over all sites. One can argue that an analysis that does not distinguish sites with different evolutionary speeds is not fair to 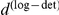. In response we put forth the following three points: (i) The main problem with 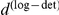 does not seem to be a lack of additivity, but rather insufficient attention to the specific types of substitution; (ii) Inhomogeneities in the evolutionary speed should affect not only the log-det distance, but most other distance measures as well. Specifically we cannot see why it should not negatively affect 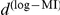 too; (iii) Similarly, analyzing sites with different speeds separately should improve the results for any distance measure – as long as it can be done unambiguously, without too much effort, and without reducing the amount of usable data excessively. In view of the last three caveats we believe that “naive” analyses, such as the one presented above, have and will continue to have their merits.

### The full picture: Comparison of several distance metrics

So far we have only compared in detail quartet classifications based on log-det distances and on single letter Shannon MI. We have used Shannon MI because its estimation is less ambiguous than compression based MI estimation, and because it uses *exactly* the same input — the base substitution frequency matrix after removing indels — as the log-det distance. But our tenet is, of course, that compression based estimates should be superior as long as they use the information about indels efficiently. In addition to the log-det distance, there are several measures that are often used. In this subsection we make several pairwise comparisons similar to the one made in the previous subsection. But we restrict ourselves to mammals, as these have the best understood phylogeny, and we expect the least numbers of errors in the literature classification.

In this subsection we compare MI based distances with the log-det and with both versions of the Kimura distance (Eqs. (S8,S9)) discussed in Material S1. We do not present all possible combinations, as this would produce a huge matrix. Instead, we focus on a subset of the distance measure pairs, but we claim that this subset is large enough to present a clear overall picture.

Results are shown in Table 2. As mentioned above, we analyzed only mammals for this, but we looked at *all* possible quartets. Our criteria for identifying the “worst” disagreements is the same as in the previous subsection. Each comparison is based on at least 60 disagreeing quartets. In this table, “Kimura
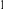
” and “Kimura
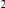
” refer to Eqs. (S8) and (S9) in Material S1, respectively; “Shannon nolog-MI” stands for 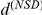 (Eq. (10)), “Shannon log-MI” stands for the logarithmic version of the Shannon distance (Eq. (11), right hand side), “transl. string, nolog” stands for 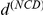 (Eq.(9) with the MI estimated via alignment), “transl. string, log” stands for its logarithmic version (Eq.(11), left hand side), “XM compression, nolog” stands for 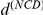 with the MI estimated via concatenation and compression with XM, and “XM compression, log” stands for its logarithmic version.

**Table 2:** Pairwise comparisons between different distance measures for complete mammalian mtDNA.

type 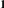	: type 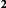	first :	second :	neither :	undecided
Shannon log-MI	: log-det	64 :	2 :	0 :	4
Shannon log-MI	: Kimura 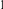	33 :	24 :	4 :	1
Shannon log-MI	: Kimura 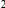	51 :	6 :	4 :	0
Kimura 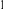	: Kimura 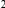	48 :	7 :	3 :	3
transl. string, nolog	: Shannon log-MI	40 :	14 :	6 :	1
XM compression, nolog	: Shannon log-MI	40 :	13 :	0 :	2
XM compression, nolog	: transl. string, nolog	52 :	12 :	0 :	1
Shannon nolog-MI	: Shannon log-MI	53 :	29 :	11 :	7
transl. string, nolog	: transl. string, log	23 :	35 :	2 :	3
XM compression, nolog	: XM compression, log	54 :	3 :	2 :	1

Compared are the abilities of 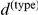 to correctly classify a large number of quartets. First, the topologies of the quartet trees obtained with two distances 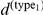 and 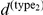 are computed. The quartets with “worst” disagreements are then looked up in the literature. Based on the literature consensus it is decided which of the two topologies is correct – unless both are wrong, or no consensus can be arrived at due to non-existent or conflicting literature. The four numbers in the columns 3 to 6 are the number of cases in which (1) the distance measure 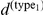 predicted the correct topology, (2) 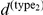 predicted the correct topology, (3) none of them did, and (4) no decision is possible.

In the present paper we have not presented any detailed application to a specific open phylogenetic problem. We also have not considered larger phylogenetic trees, in view of the imperfections of all existing distance based tree reconstruction algorithms. Instead, we have concentrated on quartets, since there we can obtain high statistics and the inference of the tree from a given distance matrix is trivial. Also, for the most detailed numerical comparison we have concentrated on Shannon information based methods, rather than on compression based methods for estimating MI. The reason is simply that we desired a comparison with other methods (mainly the log-det distance) which is as straightforward and unambiguous as possible. Indeed, it is trivial to replace Eq. (12) by Eqs. (10), (13), (14). In this way we hope to have the best chance to convince even skeptical readers that mutual information based distance measures are useful in sequence analysis.

We have also presented similar – but less complete – analyses based on large numbers of random quartets for (at least partially) compression based algorithms and have demonstrated that distances based on data compression give even better phylogenies. Indeed, from Table 2 we can draw a number of conclusions:

- All versions based on MI are better than any version not based on MI.
- Kimura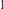 (based directly on the log-likelihood of the data with respect to the Kimura model) seems better than the conventional Kimura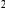, which just estimates the total number of substitutions. This supports our suspicion that counting transitions and transversions with the same weight is not a good strategy.
- Nevertheless, 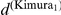 does worse than 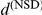, as expected: As we point out in Material S1, the log-likelihood for the Kimura model is essentially a coarse grained MI, where different substitutions are lumped together (resp., the probabilities predicted by the model replace the true observed probabilities). It would be hard to see why this should give superior results, given the ease and robustness with which single letter Shannon entropies can be estimated.
- Within the class of MI based distances, those which do not neglect indels seem systematically better.
- Among the latter, distances based on 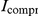 do better than those based on 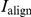. This is surprising, as we saw that 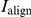 is for mammals systematically larger (and thus supposedly better) than 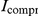.
- Logarithmic transformation of MI based distances seems to give mixed results. It improves the distances slightly for Shannon MI and for 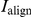, but it has very negative effects when used with 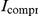 based on XM. We conjecture that this reflects two sides of the logarithmic transformation for distantly related pairs. On the one hand, it largely eliminates *systematic* errors due to deviations from metric additivity (the Felsenstein phenomenon). On the other hand it amplifies noise. To illustrate this, we discuss in Material S1 a quartet where both the original Shannon MI based distances and their log-transformed versions give wrong results, but for opposite reasons. We speculate that the detrimental effect dominates for 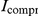, because MI estimation by compression is more noisy (due to the less systematic way that present state-of-the-art compression algorithms work) than 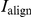.

Thus, contrary to wide spread opinion, information about indels can be directly used for phylogenetics, even without any detailed model for how they were generated. A more detailed presentation of these data and their implications will be given elsewhere.

We believe that so far we have only scratched the true potential of (algorithmic) information theory for sequence analysis. Several generalizations and improvements are feasible and are listed below:

1. Use more efficient encodings of the translation string. For instance, we only used the letters 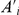 and 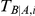 to reconstruct 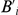, but one could also use in addition 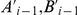, and/or 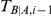.
2. Use local alignments instead of global ones. In a local alignment between sequences 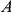 and 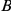, large parts of 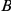 are not aligned with 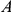 at all and are encoded without reference to 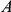. Only the aligned parts give information from 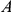 that can be used to recover 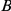. Before making the jump from global to local alignments, an intermediate step would be a “glocal” alignment tool such as shuffle-lagan (“slagan”) of [2].
3. Construct objective measures based on information theory for the quality of multiple alignments. A straightforward measure is the information about sequence 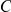 obtained from aligning it simultaneously with 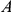 and 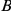. Assume e.g. that the sequences 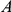 and 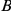 are much more similar to each other than either 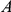 and 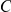 or 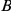 and 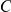 (as for human, chimpanzee, and chicken). In order to measure the MI between chicken and the primates, one could first align 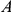 and 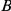 and then align, in a second step, 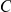 to the fixed alignment 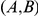.

### Conclusions

At present, biological sequence analysis is heavily based on the concept of alignment. There exist proposals for alignment-free approaches, and it has been suggested that they will become more and more important as more sequence data become available [36]. To us it seems an open question whether alignment-free algorithms for sequence comparison will become widely used, whether they will eventually displace alignment-based algorithms, or whether both approaches will merge into a unified approach. We hope that we have shown with the present work that an amalgamation of both methods (alignment-based and alignment-free) is possible. More precisely, by showing that mutual informations between two sequences can be easily estimated from global alignments, we have established a direct link between sequence alignment, Shannon information theory, and methods based on data compression and Kolmogorov information theory. Technically, we have dealt only with pairwise global alignment, but at least the basic concepts should have much wider applicability.

From another point of view, the present paper deals with the basic notion of *parsimony*. In bioinformatics (and in phylogeny in particular) maximal parsimony in dealing with several objects is often taken as synonymous to minimal number of changes needed to go from the description of one object to the description of another. This is most clearly formulated in the so-called “maximum parsimony method” of distance-free phylogenetic tree construction [7], but it also underlies the concepts of p- and log-det distances. However, the invention of the Morse alphabet in the nineteenth century, and the theoretical works by Shannon, Kolmogorov, and others in the middle of the last century might cast some doubt on it. It is Rissanen's *minimum description length principle* [37], [38], however, that makes this view obsolete today. Instead of paying attention to the *number* of changes, one should pay attention to the *information* needed to encode these changes. We call this “true parsimony”. In this sense, the maximum parsimony method does not really aim for maximal true parsimony. On the other hand, likelihood based and Bayesian methods do aim for true parsimony, but at the cost of depending on explicit models. One goal of the present paper is to show how true parsimony can be measured in less model dependent ways and how maximum true parsimony can be achieved to various degrees of approximation. Moreover, even the crudest approximation – based on MI obtained via single-letter Shannon entropies, with all information about indels discarded – can lead to important practical improvements.

## Supporting Information

Material S1 Additional figures, tables and discussion.
(2.82 MB PDF)
