# Supplementary material for: Sequence Alignment, Mutual Information, and Dissimilarity Measures for Constructing Phylogenies
Source: PLoS One. 2011 Jan 4;6(1):e14373. doi: 10.1371/journal.pone.0014373 (PMC3014950; doi:10.1371/journal.pone.0014373)
Supplement: Material S1 — Additional figures, tables and discussion. (2.82 MB PDF) [file pone.0014373.s001.pdf]

Supplementary Material

Sequence alignment, mutual information, and dissimilarity measures for constructing phylogenies

O. Penner, P. Grassberger, and M. Paczuski

1) Mutual information (MI) provides in principle an absolute and objective measure of the similarity between any two sequences A and B built from a finite alphabet. It is, however, not easily estimated – and not entirely without any model assumptions such as absence of long range correlations or optimality of compression algorithms. MI estimates can be obtained by means of Shannon information theory or via algorithmic complexity. We first discuss the latter method and turn to Shannon theory in our discussion of quartets. A main result of our paper is that MI can be estimated from alignments in general, and from *global* alignments in particular. This is done by first using an alignment between A and B to construct two 'translation strings', $T_{B|A}$ and $T_{A|B}$, which allow to reconstruct uniquely B from A and A from B, respectively. After compression, the lengths of these strings give estimates of the conditional algorithmic informations $K(B|A)$ and $K(A|B)$. Finally, the (algorithmic) MI is estimated by means of the general relations [1]

$$I(A;B) = K(A) - K(A|B), \quad I(B;A) = K(B) - K(B|A), \tag{1}$$

where $K(A)$ and $K(B)$ are the algorithmic informations (also called "Kolmogorov-Chaitin complexities") of the sequences A and B. These are estimated by the lengths of compressed versions of A and B. A central result of algorithmic information theory is that $I(A;B) = I(B;A)$ up to terms $O(\log N)$, where N is the length of the concatenation AB [1].

The mutual informations estimated this way, denoted $I_{\rm align}$ (see Eq. (3)), can be compared to estimates of MI obtained without using any alignment. The latter can be obtained by comparing the combined lengths of the compressed versions of A and B to the length of a compressed version of the concatenation AB. We denote this $I_{\rm compr}$ and it is given by Eq. (4). An unexpected result of the present paper is that both estimates, in spite of being independent and following rather different strategies, yield very similar results for mitochondrial DNA (mtDNA) of vertebrates. More precisely, they give practically *identical* estimates for species within the same family. $I_{\rm align}$ is typically slightly larger for species in the same class but in different families, while $I_{\rm compr}$ is larger for species in different phyla, a case where global alignment algorithms break down.

2) The estimate $I_{\rm compr}$ is the standard estimate for the MI between two strings, and has been used recently in a large number of biological and non-biological problems [2–6]. Most of the work done with $I_{\rm compr}$ has focused on

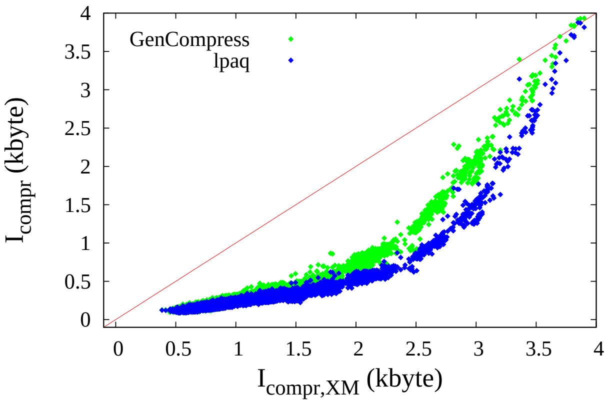

FIG. 1: Scatter plot comparing MI estimates $I_{\rm compr}$ obtained with different compression algorithms, for complete mammalian mtDNA. Note that XM produces higher and, hence, better estimates of $I_{\rm compr}$ than both GenCompress and lpaq1. In addition to lpaq1 we also tested dark, bbb, paq9, paq8, and durilca [9]. The results for all of these algorithms are qualitatively the same as those shown for lpaq1.

clustering of sequences and building phylogenetic trees, however it seems that this activity has met with considerable skepticism in the biological community. One reason is that it seems as if no biological knowledge is incorporated to estimate $I_{\rm compr}$. This is in stark contrast to the substantial amount of biological exertise that goes into constructing phylogenies based on alignments. Another concern is that the value of $I_{\rm compr}$ depends on the quality of the compression algorithm. While two compression algorithms can be easily compared via the lengths of the compressed files they produce, it is impossible to judge the quality of a compression algorithm on an absolute scale. Most non-trivial sequences like DNA, proteins, music, or written language show long range and structurally complex correlations. To obtain good estimates of $I_{\rm compr}$ it is crucial to take these long range effects into account. However, their precise structure and their effect on compressibility are usually not known.

A last reason why compression based methods in phylogeny have been viewed critically is that the MI-based distance measures used in [2–6] are not *additive* (or "tree metrics"), i.e. they do not satisfy the four-point condition [7]. According to [3, 4], the preferred distance is the *normalized compression distance* $d^{\rm (NCD)}$ defined in Eq.(9). Instead of increasing linearly with evolutionary distance, it tends to $d^{\rm (NCD)} = 1$ for maximally distinct pairs. This is considered a severe drawback in phylogenetic applications, in particular it leads to the phenomenon of 'long branch attraction' [8].

It was accepted, up to the turn of the century, that biosequences (DNA, amino acids) are hardly compressible [10, 11], and work on special compression algorithms for DNA and amino acid sequences began. Although there has been substantial progress during the decade in

general purpose compression algorithms, we found that still none of the general purpose algorithms we checked (lpaq1, durilca, bbb, dark [9]) gave better compression for DNA than GenCompress [2] – one of the best public domain DNA compression algorithm available prior to 2007. But the latter also gives poor compression rates, and it was not clear whether this is an inherent problem of DNA or due to weaknesses of the algorithms.

The situation has changed substantially with the development of XM [12], which gives unambigously better results than previous algorithms. According to the authors of Ref. [12], the improvement brought by XM over previous DNA compression algorithms (such as GenCompress) is only a few per cent, when single sequences are considered. However, we found that the improvement in estimates of $I_{\rm compr}$ is much larger for concatenated pairs, presumably because long range correlations and approximate repetitions – which are better captured by XM – play a much larger role.

Results for $I_{\rm compr}$ using GenCompress and lpaq1 versus $I_{\rm compr}$ using XM are shown in Fig. S1. We observe vast differences, except for very closely related species, where all three compression algorithms detect the strong similarity. For species in different families, XM gives typically three to five times larger MIs than GenCompress. Note that we do not have a rigorous proof that larger values of $I_{\rm compr}$ are more accurate. The difficulty is that $I_{\rm compr}$ is the difference between terms which are all overestimated. But the negative term, corresponding to $K(AB)$, is the most difficult to estimate and hence the most likely to be strongly overestimated. As such, it follows that larger values of $I_{\rm compr}$ indicate improved treatment of long-range interdependencies and better MI estimates.

The very strong dependence on compression algorithm observed in Fig. S1 seems to justify skepticism against the use of $I_{\rm compr}$. This is contradicted, however, by the fact that the values of $I_{\rm compr}$ obtained with XM are in very good general agreement with the values of $I_{\rm align}$, as shown in Fig. 2 of the main paper. The latter suggests that it should be possible to improve $I_{\rm compr}$ further by a factor ≈ 1.5 for species in different classes and by ≈ 2 for species in different phyla, but probably not by more.

3) As pointed out in the main text, one can estimate the conditional complexity of a sequence B, conditioned on another sequence A, by simply compressing the translation string $T_{B|A}$. This would give Eq.(1), and it would be the best possible estimate if $T_{B|A}$ were independent A. This is certainly a reasonable first approximation, but to check it we estimated the MI between $T_{B|A}$ and A for a large number of mtDNA pairs. Notice that this MI is precisely – up to corrections of order $\log(N)$ and up to the uncertainties of estimating MI using a given compression algorithm – the difference between Eqs.(2) and (1). Results are shown in Fig. S2. They show that correlations between translation and conditioning sequences are in all cases small and could have been neglected without introducing large errors. They have a tendency to

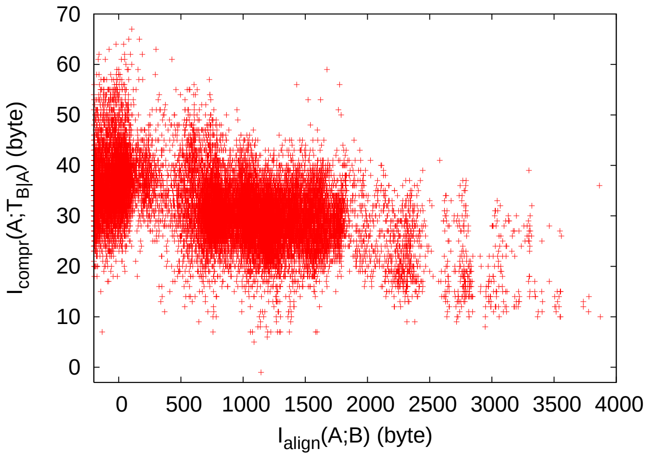

FIG. 2: Scatter plot of mutual informations between translation strings $T_{B|A}$ and conditioning strings A, versus the MI between A and B. Both values are estimated via compression with lpaq1. The plot uses the same mtDNA pairs as Figure 2.

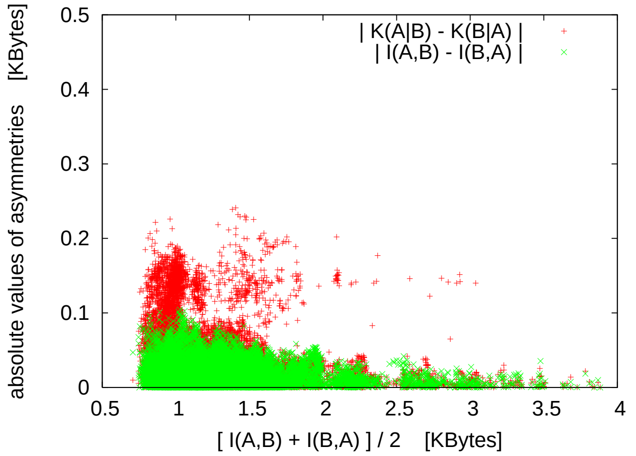

FIG. 3: Scatter plot of asymmetries of $I_{\rm align}$ and of conditional informations, both versus $I_{\rm align}$. In contrast to the previous figures, this plot involves only mammals.

decrease with increasing $I_{\rm align}(A;B)$.

4) An important aspect of our treatment is that the translation strings $T_{B|A}$ and $T_{A|B}$ are different, as long as they contain indels. Thus the conditional informations $K(B|A)$ and $K(A|B)$ are also different. This is in stark contrast to the notion of edit distances, where one typically defines a symmetric distance measure directly via the number and costs of edit operations. In our case the symmetric distance is obtained by first estimating MIs and then by deriving compression distances from them [3]. Although this appears more complicated, it has the advantage of providing a direct link with information theory.

A crucial requirement for our formalism is that the estimates $I_{\rm align}$ should be symmetric under the exchange of the sequences within terms of $O(\log N)$, $I(A;B)_{\rm align} \approx$

```
A     GGGAGATATAGCCGTTATGTGAATAGCACTGAATCAAGCGGCGTACGCGTTATGTGA
B

A'    GGGAGATATAGCCGTTATGTGAATAGCACTGAATCAAGCGGCGTACGCGTTATGTGA
B'    ---------------------------------------------------------
TA|B  GGGAGATATAGCCGTTATGTGAATAGCACTGAATCAAGCGGCGTACGCGTTATGTGA
TB|A  ---------------------------------------------------------
```

FIG. 4: Alignment and translation strings for comparing a random sequence with an empty one. Here, sequence A is random of length n, while B is empty. As explained in the text, the estimated MIs $I(B;A)_{\rm align}$ and $I(A;B)_{\rm align}$ agree with the expected results within terms of order $\mathcal{O}(\log n)$.

```
A     GGGCGCTCTCGAAGTTCTGTGCCTAGTT
B     CCCCCCCCCCCCCCCCCCCCCCCCCCCC

A'    GGGCGCTCTCGAAGTTCTGTGCCTAGTT----------------------------
B'    ----------------------------CCCCCCCCCCCCCCCCCCCCCCCCCCCC
TA|B  GGGCGCTCTCGAAGTTCTGTGCCTAGTT----------------------------
TB|A  ----------------------------CCCCCCCCCCCCCCCCCCCCCCCCCCCC
```

FIG. 5: Alignment and translation strings for comparing a random sequence with a sequence composed of only one letter. Here, A is a random sequence of length n, while B is a string of n letters "C". As explained in the text, the estimated MIs $I(B;A)_{\rm align}$ and $I(A;B)_{\rm align}$ again agree with the expected results within terms of order $\mathcal{O}(\log n)$.

$I(B;A)_{\rm align}$. Any strong violation of this symmetry would indicate that either the construction of the translation string is not optimal, or that the compression algorithm used in Eq. (3) is deficient. In contrast it is not required that $K(B|A)$ is symmetric. To show that $I(A;B)_{\rm align}$ is more symmetric than $K(B|A)$, we plot in Fig. S3 the differences $|I(A;B)_{\rm align} - I(B;A)_{\rm align}|$ and $|K(B|A) - K(A|B)|$ against $I(A;B)_{\rm align}$. Here we consider only mammalian mtDNA, because the estimates $I(A;B)_{\rm align}$ for species in different classes are too uncertain for a meaningful analysis. We see that there are no problems at all for closely related species, as in such cases both $I(A;B)_{\rm align}$ and $K(B|A)$ are symmetric. For more distant species, both are still symmetric for the majority of pairs, but there are also numerous outliers where $K(B|A)$ is strongly asymmetric. In all those cases the asymmetry of $I(A;B)_{\rm align}$ is significantly smaller than that of $K(B|A)$.

5) In the main text we argued how the symmetry of $I(A;B)_{\rm align}$ is compatible with asymmetric values of $K(B|A)$. Here we shall present some exact results for some extreme cases.

a) Assume that string A is a random string, of length n, over the alphabet $\{A,C,G,T\}$ and B is the empty string. Then the optimal alignment is shown in Fig. S4. In order to specify $T_{B|A}$ one must encode the letter "-" and the number n of repetitions, giving $K(B|A) \approx \log_2 n$ bits. On the other hand, $K(A|B) = K(A)$. These give $I(B;A)_{\rm align} \approx -\log_2 n$ bits and $I(A;B)_{\rm align} = \mathcal{O}(1)$. The reason why $I(A;B)_{\rm align}$ is not exactly zero is that the two terms on the r.h.s. of Eq. (3) are compressed using different algorithms. Both have to be specified verbatim, so the only difference between the two term in Eq. (3) is the difference between the overheads in lpaq1 and XM.

```
      A     CCCTCCCCGCCCACCCCTCCCCCGCCAA
      B     CCCCCCCCCCCCCCCCCCCCCCCCCCCC

                  Alignment 1
      A'    CCCTCCCCGCCCACCCCTCCCCCGCCAA
      B'    CCCCCCCCCCCCCCCCCCCCCCCCCCCC
      TA|B  0001000030002000010000030022
      TB|A  0001000030002000010000030022

                  Alignment 2
A'    CCCTCCCCGCCCACCCCTCCCCCGCCAA----------------------------
B'    ----------------------------CCCCCCCCCCCCCCCCCCCCCCCCCCCC
TA|B  CCCTCCCCGCCCACCCCTCCCCCGCCAA----------------------------
TB|A  ----------------------------CCCCCCCCCCCCCCCCCCCCCCCCCCCC
```

FIG. 6: Two alternative alignments between a biased random sequence and a sequence composed of only one letter. See the text for details.

In summary, $I(B;A)_{\rm align} - I(A;B)_{\rm align} = O(\log n)$, as expected on general grounds for the difference between the exact MI values.

b) Assume that A is as in the above case, and B a string of the same length consisting of a single letter, say "C". The optimal alignment for this case is shown in Fig. S5. Now $T_{B|A}$ consists of n hyphens followed by n "C"s, which gives $I(B;A)_{\rm align} \approx -\log_2 n$ bits. Similarly, $T_{A|B} = A$, followed by n hyphens, so that also $I(A;B)_{\rm align} \approx -\log_2 n$. Thus, for Fig. S5 the difference $I(B;A)_{\rm align} - I(A;B)_{\rm align}$ is again as expected for the exact MI values.

c) Finally, we can consider a situation similar to case b), but with A not fully random. Instead, we assume that A is iid. with $\text{prob}(A_i = \text{C}) \gg \text{prob}(A_i = \text{A}) = \text{prob}(A_i = \text{G}) = \text{prob}(A_i = \text{T})$. In this case one might be inclined intuitively to prefer alignment #1 of Fig. S6 over alignment #2. But for alignment #1 both $T_{B|A}$ and $T_{A|B}$ would be equal to A, up to a complexity preserving transformation A$\to$1, C$\to$0, G$\to$3, and T$\to$2. Thus $I(B;A)_{\rm align} \propto n$, while $I(A;B)_{\rm align} = \mathcal{O}(1)$. On the other hand, for alignment #2 both $I(B;A)_{\rm align}$ and $I(A;B)_{\rm align}$ are the same as in Fig. S5. Thus alignment #2 is the optimal alignment, although one might have preferred alignment #1 intuitively.

6) Up to now, we have assumed that the two DNA files contain only the four letters "A", "C", "G", and "T". In reality, the data banks allow also for "wild card" letters indicating ambiguities: "N" for any nucleotide, "R" for a purine, "Y" for a pyrimidine, etc. [13]. Whenever either of the two sequences contains such a wild card character, we put $T_{B|A,i} = B_i$, i.e. the letter in the target string is copied verbatim. This will in some cases slightly overestimate the conditional information $K(B|A)$. But such overdestimations are expected to occur anyhow, and in the data base we used, wild cards are sufficiently rare to have very little effect.

7) In the case of proteins (i.e. amino acid sequences) we have an alphabet of 20 letters. This makes the analysis a bit more lengthy, although it is otherwise the same as for DNA. The translation strings now contains 41 characters: Twenty letters for specifying insertions, one hyphen for indicating a deletion, and 20 numbers for indicating

```
A'      GGGAG---ATATAGCCGTTATGTGAATAGCACTGAATCAAGCG---------GCGT
B'      GGGAC---ATATAG----TATGTAAATATCACTGAATCGAACGAACACGCACGTGT
C'      GGGACGGGATATAGCCGTTATGTAA-----ACTGTATCAAGCGAACACGCACGTGT
T_C|A,B 00003GGG000000C0000000010-----00003000100000A000000000000
```

FIG. 7: Alignment of a third sequence C to an already existing alignment between A and B. The translation string $T_{C|AB}$ for reconstructing C from A and B is obtained by using locally one of the strings A and B as "master strings" and applying the rules described in the main part of the paper. The actual master string is printed in red.

substitutions. For the latter we have some freedom. As for DNA, we could encode forward and backward substitutions by the same number. Apart from that, we could use this freedom in the case of amino acids also to optimally encode substitutions. This optimization would likely rely on PAM or similar matricies. Alternatively, we could encode the 20 amino acids by numbers $0 \dots 19$, and encode a substitution $j \to k$ ($j, k = 0 \dots 19$) by the number $k - j \mod 20$. This has the advantage of simplicity, but led in the case of DNA to marginally worse results.

8) For local alignments, the output of an alignment algorithm consists of a list of matching regions, together with the actual alignments of those regions. These matching regions can in principle overlap. Thus in order to construct $T_{B|A}$, one first has to select a subset of matching regions $\mathcal{M}_k$ that do not overlap on sequence B. Each one of these matches is characterized by its starting points in sequences A and B and by the translation string restricted to the matching regions,

$$\mathcal{M}_k \equiv \{n_k^{(A)}, n_k^{(B)}, T_{B|A;k}\}. \tag{2}$$

The entire translation string consists of all these pieces of information, separated by uniquely decodable separators, plus the verbatim description of sequence B in regions where no matches were found or used. Indeed, if one has the latter verbatim description and all translation strings $T_{B|A;k}$ for the matching regions, one can recover the points $n_k^{(B)}$ and does not have to include them in $T_{B|A}$ explicitly.

9) As a first step towards multiple alignments we discuss the alignment of a sequence C to an already existing and fixed alignment of two sequences A and B, and the corresponding MI estimate $I(C;(A,B))_{\rm align}$. An application of this could be to estimate the similarity between mouse and the family of hominides, where the latter is characterised by the two species homo and chimpanzee. The central problem is to construct a translation string which allows to reconstruct C from the aligned pair (A, B).

One possibility is illustrated in Fig. S7. We start at position $i = 1$ and construct $T_{C|AB}$ from left to right, using at each step one of the sequences A or B as the "master sequence". If A is presently the master sequence, then $T_{C|AB,i} = T_{C|A,i}$, where $T_{C|A,i}$ is as described in the main paper. Similarly, if B is the master sequence, then $T_{C|AB,i} = T_{C|B,i}$. The status of master sequence is kept until C'_i disagrees with the character in the master sequence *and* is identical to the character in the other (non-master) sequence, at which point master and non-master sequences switch their status. In a slightly more sophisticated version, one keeps track of the number of 'mistakes' made recently by sequences A and B, and switches only when the current master has done worse over the recent past. We have no proof that either encoding is optimal, but both guarantee at least that the "better" of the two sequences A and B is used as a template to reconstruct C.

We note estimating $I(C;(A,B))$ via concatenation and compression alone is easy. One just has to modify Eq. (4) to

$$I(C;(A,B))_{\rm compr} = {\rm len}[{\rm XM}(AB)] + {\rm len}[{\rm XM}(C)] - {\rm len}[{\rm XM}(ABC)], \tag{3}$$

where AB and ABC denote the concatenations of A with B and of AB with C. This gives $I(C;(A,B))_{\rm compr} \geq I(C;A)_{\rm compr}$ and $I(C;(A,B))_{\rm compr} \geq I(C;B)_{\rm compr}$, in agreement with general relations between MIs and with intuition. In contrast, concatenating A and B and then aligning (globally!) C with AB would lead to very poor estimates of $I(C;(A,B))_{\rm align}$.

We have made preliminary numerical tests showing that the proposals in points 6) to 8) are potentially feasible, but more complete investigations are needed and will be presented in future publications.

10) **Definition of significances for quartets**

For any quartet of sequences A, B, C and D, we first measure the distance matrix d_{ij} with $i, j \in \{A, B, C, D\}$. If these distances satisfy the four-point condition, we obtain

$$b \equiv \frac{1}{2} \max\min\{d_{ij} + d_{kl} - d_{ik} - d_{jl}, d_{ij} + d_{kl} - d_{il} - d_{jk}\} = 0, \tag{4}$$

where the maximum is over all permutations $(A, B, C, D) \to (i, j, k, l)$ and the minimum is, for each permutation, over the two terms in the curly bracket. If $b > 0$, the four-point condition is violated. A second quantity of interest is

$$a \equiv \frac{1}{2} \max(d_{ij} + d_{kl} - d_{ik} - d_{jl}) \,. \tag{5}$$

Where the maximum is over permutations of the indicies. Obviously, $b \leq a$.

If $b = 0$, i.e. if the distances can be exactly represented by an (unrooted) tree, then a is the length of the central edge. If not, the distances cannot be represented by a tree, but by the most simple example of a *split graph* [14]: A graph consisting of a parallelepiped in the center and four legs connecting to the leaves A to D. The two quantities a and b are the lengths of the two edges of the parallelepiped. If $b = a$ the inner box is a rhombus

and no partitioning of the quartet into pairs $((ij)(kl))$ is preferred. Thus we take $a-b$, after normalizing it properly, as a significance measure,

$$S = \frac{2(a-b)}{\max\{d_{ik}+d_{jl}, d_{il}+d_{jk}\}} \quad . \tag{6}$$

The preferred partition is that for which $d_{ik}+d_{jl}-d_{ij}-d_{kl} > 2b$. The significance is bounded by $0 \leq S \leq 1$.

11) **Most significant disagreements for topologies based on $d^{(\log-\mathrm{det})}, d^{(\log-\mathrm{MI})}$, and $d^{(\mathrm{NSD})}$**

If two metrics predict different topologies 1 and 2 with significances S_1 and S_2, then we define the significance of the disagreement as $S_{12} = S_1 + S_2$. A list of quartets that contain some of the most significant clashes between $d^{(\log-\mathrm{det})}$ and $d^{(\log-\mathrm{MI})}$ are found in Tables S1 to S9, with the somewhat unsystematic selection criteria discussed in the main text. We should stress, however, that the selection – which was made to minimize clustering of quartets in few taxa – was done blindly, i.e. by representing species by numbers and avoiding triplets that occur more than once in the list.

Clashes between $d^{(\log-\mathrm{MI})}$ and $d^{(\mathrm{NSD})}$ were fewer – less than half of those between $d^{(\log-\mathrm{det})}$ and $d^{(\log-\mathrm{MI})}$ – and had smaller significances. The largest values of S_{ij} were typically only half of the largest S_{ij} for $d^{(\log-\mathrm{det})}$ and $d^{(\log-\mathrm{MI})}$, for each clade. The smaller significances together with generally poor agreement in the literature, as to the "true" groupings, suggests that these clashes are of less biological significance, and we do not list them.

12) **Definition of two distance measures based on the 2-parameter Kimura model**

In Kimura's model [15] it is assumed that transitions (A↔ G, C↔T) and transversions (A↔ C, G↔T,A↔ T, C↔G) have different rates α and β. For fixed α, β, and evolution time t, the log-likelihood for observed substitution frequencies $f_{AB}(i,k)$ is [16] (p. 230)

$$L_{AB} = m_{\mathrm{ident}} \log r + m_{\mathrm{transi}} \log s + m_{\mathrm{transv}} \log u, \tag{7}$$

where m_{type} is the number of observed types of "substitutions", $s = (1-e^{-4\beta t})/4$, $u = (1-e^{-4\beta t} - 2e^{-2(\alpha+\beta)t})/4$, and $r = 1-2s-u$. Notice that when we assume β and α are drawn from a model (as opposed to fitting), this is reminiscent of a Kullback-Leibler [1] version of MI,

$$L_{AB} = \frac{1}{M} \sum_{i,j\in\{A,C,G,T\}} f_{AB}(i,j) \log \frac{Q_{ij}}{Q_i Q_j} - \log 4.$$

Here M is the length of the sequences, $\mathbf{f}_{AB}$ is the observed substitution matrix, $\mathbf{Q}$ is the substitution matrix of the model,

$$\mathbf{Q} = \begin{pmatrix} r & s & u & s \\ s & r & s & u \\ u & s & r & s \\ s & u & s & r \end{pmatrix} \quad , \tag{8}$$

and the equilibrium distribution of the model is $\vec{Q} = (1/4, 1/4, 1/4, 1/4, 1/4)$. For given m_{type}, the likelihood is maximal if $u = m_{\mathrm{transi}}$, $s = m_{\mathrm{transv}}/2$, and $r = m_{\mathrm{ident}}$, which in turn gives values for αt and βt.

The most natural *a priori* candidate for a distance in the Kimura model is the absolute value of L itself,

$$d_{AB}^{(\mathrm{Kimura}_1)} = -L_{AB}. \tag{9}$$

This is, however, *not* what is commonly used. Rather, the *Kimura distance* is defined as the average total number of substitutions, including the "hidden" ones, which is

$$\begin{aligned} d_{AB}^{(\mathrm{Kimura}_2)} &= (\alpha+2\beta)t = -\frac{1}{2}\log\frac{m_{\mathrm{ident}} - m_{\mathrm{transi}}}{M} \\ &- \frac{1}{4}\log\frac{m_{\mathrm{ident}} + m_{\mathrm{transi}} - m_{\mathrm{transv}}}{M}. \end{aligned} \tag{10}$$

Although this distance has the advantage of being additive, it has serious drawback: Given that transitions and transversions occur at different rates, it does not seem to make much sense to taken an unweighted sum over both as a distance measure.

13) **An example of quartet misclassification due to metric non-addivity**

We discuss here the quartet formed by three Ursus species (*U. maritimus, U. americanus*, and *U. thibetanus*) and a Lagomorph species (*Orcytolagus cuniculus*). According to [41], the grouping of the three Ursidae is ((*maritimus, americanus*), *thibetanus*). The two distance matrices obtained with $d^{(\mathrm{NSD})}$ and with $d^{(\log-\mathrm{MI})}$ (i.e. with the original and the log-transformed versions of the Shannon-MI based distances) are shown in Table 10. We see that the smallest distance is between *U. americanus* and *U. thibetanus* Hence, the evolutionary speeds within the Ursidae must have been different. Using $d^{(\mathrm{NSD})}$, the outgroup species (*O. cuniculus*) has roughly the same distances from all three (because they are already close to saturation, as expected for a distant outgroup and a metric where distances converge to a finite value when evolutionar distances tend to infinity), and thus the Felsenstein phenomenon gives the wrong grouping ((*americanus, thibetanus*), *maritimus*). What is no longer true after log-transforming, as this increases the distance differences to the outgroup species. But since these differences are affected by amplified random errors, the resulting grouping ((*maritimus, thibetanus*), *americanus*) is also wrong.

Note that this example is better called "small distance attraction", in contrast to the "large distance attraction" observed in standard textbook examples of the Felsenstein phenomenon. The latter is due to the fact that in these examples *two* species are distant from the other two, while in our example only one is distant from the other two. But the basic reason for the misclassification is the same, and "large distance" attraction is indeed at the same time also a "small distance" attraction. We chose our example for more intuitive interpretation.

[1] T. Cover and J. Thomas, *Elements of information theory* (John Wiley & Sons, Hoboken, 2006), 2nd ed.
[2] M. Li, J. Badger, X. Chen, S. Kwong, P. Kearney, and H. Zhang, Bioinformatics **17**, 149 (2001).
[3] M. Li, X. Chen, X. Li, B. Ma, and P. Vitanyi, IEEE Trans. Inf. Theory **50**, 3250 (2004).
[4] R. Cilibrasi and P. Vitanyi, IEEE Trans. Inf. Theory **51**, 1523 (2005).
[5] A. Kraskov, H. Stoegbauer, R. Andrzejak, and P. Grassberger, Europhysics Letters **70**, 278 (2005).
[6] A. Kraskov and P. Grassberger, *Mic: Mutual information based hierarchical clustering* (2008).
[7] W. Press, S. Teukolski, W. Vetterling, and B. Flannery, *Numerical Recipes, 3rd edition* (Cambridge University Press Cambridge, 2007).
[8] J. Felsenstein, J. Mol. Evol. **17**, 368 (1981).
[9] *http://cs.fit.edu/~mmahoney/compression/text.html* (2008).
[10] S. Grumbach and F. Tahi, DCC pp. 340–350 (1993).
[11] *http://monod.uwaterloo.ca/downloads/gencompress/* (2003).
[12] M. Cao, T. Dix, L. Allison, and C. Mears, Proceedings of the 2007 Data Compression Conference pp. 43–52 (2007).
[13]
[14] H. Bandelt and A. Dress, Molecular Phylogenetics and Evolution **1**, 242 (1992).
[15] M. Kimura, Journal of Molecular Evolution **16**, 111 (1980).
[16] R. Durbin, S. Eddy, A. Krogh, and G. Mitchison, *Biological sequence analysis: Probabilistic models of proteins and nucleic acids* (Cambridge University Press Cambridge, 1998).
[17] Y. Kitazoe, H. Kishino, P. Waddell, N. Nakajima, T. Okabayashi, T. Watanabe, and Y. Okuhara, PLoS one **2**, e384 (2007).
[18] G. Wesley-Hunt and J. Flynn, Journal of Systematic Palaeontology **3**, 1 (2005).
[19] J. Flynn, J. Finarelli, S. Zehr, J. Hsu, and M. Nedbal, Systematic Biology **54**, 317 (2005).
[20] J. Nelson, *Fishes of the World* (John Wiley, New York, 2006), 4th ed.
[21] C. Li, Ph.D. thesis, University of Nebraska (2007).
[22] W. Chen, C. Bonillo, and G. Lecointre, Molecular Phylogenetics and Evolution **26**, 262 (2003).
[23] P. Gaubert, G. Denys, and T. Oberdorff, Biological Reviews **84**, 653 (2009).
[24] *http://tolweb.org/Neoptera/8267* (2003).
[25] *http://www.ncbi.nlm.nih.gov/Taxonomy/Browser/wwwtax.cgi?mode=Und*.
[26] S. Cameron, C. Lambkin, S. Barker, and M. Whiting, Systematic Entomology **32**, 40 (2006).
[27] *http://en.wikipedia.org/wiki*.
[28] T. Engstrom, H. Shaffer, and W. McCord, Syst. Biol. **53**, 693 (2004).
[29] R. Pratt, G. Gibb, M. Morgan-Richards, M. Phillips, M. Hendy, and D. Penny, Mol. Biol. Evol. **26**, 313 (2009).
[30] S. Eo, Zoologica Scripta **38**, 465 (2009).
[31] *http://tolweb.org/Caudata/14939* (2006).
[32] R. Mueller, J. Macey, M. Jaekel, D. Wake, and J. Boore, Proc. Natl. Acad. Sci. U.S.A. **101**, 13820 (2004).
[33] A. Jex, R. Hall, T. Littlewood, and R. Gasser, Nucleic Acids Research **38**, 522 (2010).
[34] Y. Liu and Z. Cui, Mol. Biol. Rep. pp. 10.1007/s11033–009–9773–2 (2009).
[35] N. Puillandre, S. Samadi, M. Boiselier, A. Sysoev, Y. Kantor, C. Cruaud, A. Couloux, and P. Bouchet, Molecular Phylogenetics and Evolution **47**, 1122 (2008).
[36] C. Grande, J. Templado, and R. Zardoya, BMC Evol Biol. **8**, 61 (2008).
[37] R. Cunha, C. Grande, and R. Zardoya, BMC Evol Biol. **9**, 210 (2009).
[38] A. Lavikainen, V. Haukisalmi, M. Lehtinen, H. Henttonen, A. Oksanen, and S. Meri, Parasitology **135**, 1457 (2008).
[39] E. Hohberg, Parasitology International **55**, S23 (2006).
[40] M. Nakao, D. McManus, P. Schantz, P. Craig, and A. Ito, Parasitology **134**, 713 (2006).
[41] M. Pagès, S. Calvignac, C. Klein, M. Paris, S. Hughes, and C. Hanni, Molecular Phylogenetics and Evolution **47**, 73 (2008).

mammals										
	species names						classification predicted by			
Nr.	species 1	species 2	species 3	species 4	"true"	Ref.	$d^{(\log-\mathrm{MI})}$	$d^{(\log-\det)}$	$d^{(\mathrm{NSD})}$	$S_{(\log-\det,\log-\mathrm{MI})}$
1	Ursus thibet. thibetanus	Ursus thibetanus	Ursus thibet. mupinensis	Ursus maritimus	?	–	(12)(34)	(14)(23)	(12)(34)	0.05719
2	Cricetulus griseus	Choloepus didactylus	Manis tetradactyla	Hippopotamus amphibius	(12)(34)	[17]	•	(13)(24)	•	0.03671
3	Cricetulus griseus	Pongo abelii	Manis tetradactyla	Hippopotamus amphibius	(12)(34)	[17]	•	(13)(24)	•	0.03433
4	Erignathus barbatus	Ailuropoda melanoleuca	Nyctereutes procyonoides	Mammuthus primigenius	(12)(34)	[17, 18]	•	(13)(24)	•	0.03404
5	Tremarctos ornatus	Callorhinus ursinus	Nyctereutes procyonoides	Phascogale tapoatafa	(12)(34)	[17, 18]	•	(14)(23)	•	0.03343
6	Spilogale putorius	Ceratotherium simum	Hemiechinus auritus	Ochotona princeps	(12)(34)	[17]	•	(13)(24)	•	0.03334
7	Canis lupus chanco	Physeter catodon	Hemiechinus auritus	Pongo pygmaeus	(12)(34)	[17]	•	(13)(24)	•	0.03305
8	Elaphodus cephalophus	Equus caballus	Hemiechinus auritus	Pongo abelii	(12)(34)	[17]	•	(14)(23)	•	0.03240
9	Ursus thibet. mupinensis	Phocarctos hookeri	Acinonyx jubatus	Balaenoptera omurai	(12)(34)	[17, 18]	•	(14)(23)	•	0.03220
10	Elaphodus cephalophus	Kogia breviceps	Martes zibellina	Erinaceus europaeus	(12)(34)	[17]	•	(14)(23)	•	0.03211
11	Hydropotes inermis	Neophoca cinerea	Hemiechinus auritus	Pongo pygmaeus	(12)(34)	[17]	•	(13)(24)	•	0.03199
12	Martes flavigula	Ursus thibet. mupinensis	Capricornis crispus	Spilogale putorius	(12)(34)	[18, 19]	•	(14)(23)	•	0.03191
13	Martes zibellina	Ursus thibet. mupinensis	Acinonyx jubatus	Rhinoceros unicornis	(12)(34)	[18, 19]	•	(13)(24)	•	0.03173
14	Muntiacus crinifrons	Hippopotamus amphibius	Ailuropoda melanoleuca	Ceratotherium simum	(12)(34)	[17]	•	(13)(24)	•	0.03171
15	Manis tetradactyla	Equus caballus	Hemiechinus auritus	Pongo pygmaeus	(12)(34)	[17]	•	(14)(23)	•	0.03169
16	Balaenoptera brydei	Manis tetradactyla	Hemiechinus auritus	Homo sapiens	(12)(34)	[17]	•	(14)(23)	•	0.03159
17	Martes flavigula	Tremarctos ornatus	Stenella attenuata	Canis latrans	(12)(34)	[18, 19]	•	(14)(23)	•	0.03159
18	Canis lupus laniger	Eschrichtius robustus	Hemiechinus auritus	Pongo pygmaeus	(12)(34)	[17]	•	(13)(24)	•	0.03156
19	Martes zibellina	Ursus thibet. thibetanus	Canis lupus chanco	Rhinolophus formosae	(12)(34)	[18, 19]	•	(13)(24)	•	0.03105
20	Martes zibellina	Helarctos malayanus	Canis latrans	Pteropus scapulatus	(12)(34)	[18, 19]	•	(13)(24)	•	0.03100
21	Odobenus rosmar. rosmarus	Eumetopias jubatus	Macroscelides proboscideus	Halichoerus grypus	(12)(34)	[18, 19]	•	(13)(24)	•	0.03086
22	Martes zibellina	Melursus ursinus	Mammut americanum	Felus catus	(12)(34)	[18, 19]	•	(14)(23)	•	0.02779
23	Kogia breviceps	Muntiacus reevesi	Cuon alpinus	Gorilla gorilla	(12)(34)	[17]	•	(14)(23)	•	0.02640

TABLE I: Quartets of mammals for which $d^{(\log-\mathrm{MI})}$ and $d^{(\log-\det)}$ give significantly different classifications. In the last four columns, a bullet means that the correct grouping was predicted with this distance measure; otherwise the predicted grouping is given. Within each quartet, species are arranged so that the the first two are closest according to established taxonomy (whenever available). Quartets are ordered by decreasing significance of the disagreement between log-det and log-MI groupings.

actinopterygii (ray-finned fish)										
	species names						classification predicted by			
Nr.	species 1	species 2	species 3	species 4	“true”	Ref.	$d^{(\log-\mathrm{MI})}$	$d^{(\log-\det)}$	$d^{(\mathrm{NSD})}$	$S_{(\log-\det,\log-\mathrm{MI})}$
1	Bathygadus antrodes	Pterocaesio tile	Anguilla interioris	Albula glossodonta	(12)(34)	[20–22]	•	(14)(23)	•	0.05574
2	Bathygadus antrodes	Thunnus alalunga	Anguilla obscura	Albula glossodonta	(12)(34)	[20–22]	•	(14)(23)	(14)(23)	0.05296
3	Bathygadus antrodes	Caranx melampygus	Anguilla bicolor bicolor	Albula glossodonta	(12)(34)	[20–22]	•	(14)(23)	•	0.05276
4	Bathygadus antrodes	Crenimugil crenilabis	Anguilla japonica	Albula glossodonta	(12)(34)	[20–22]	•	(14)(23)	•	0.05250
5	Bathygadus antrodes	Lophius litulon	Anguilla celebesensis	Albula glossodonta	(12)(34)	[20–22]	•	(14)(23)	•	0.05226
6	Bathygadus antrodes	Ostichthys japonicus	Anguilla dieffenbachii	Albula glossodonta	(12)(34)	[20–22]	•	(14)(23)	•	0.05200
7	Bathygadus antrodes	Dactyloptena tiltoni	Anguilla mossambica	Albula glossodonta	(12)(34)	[20–22]	•	(14)(23)	•	0.05109
8	Bathygadus antrodes	Lates calcarifer	Anguilla obscura	Albula glossodonta	(12)(34)	[20–22]	•	(14)(23)	•	0.05096
9	Spratelloides gracilis	Clupea pallasii	Ilisha elongata	Takifugu ocellatus	(12)(34)	[20]	(14)(23)	•	(14)(23)	0.03573
10	Albula glossodonta	Elops hawaiensis	Nansenia ardesiaca	Galaxias maculatus	(12)(34)	[20, 21]	•	(14)(23)	•	0.03529
11	Ceratias uranoscopus	Eutaeniophorus festivus	Cyema atrum	Gonorynchus greyi	(12)(34)	[20, 21]	•	(14)(23)	•	0.03277
12	Sprattus sprattus	Spratelloides delicatulus	Ilisha africana	Anguilla malgumora	(12)(34)	[20]	(13)(24)	•	(13)(24)	0.03236
13	Clinocottus analis	Fundulus diaphanus	Salangichthys microdon	Puntius ticto	(12)(34)	[20]	•	(13)(24)	•	0.03114
14	Diplacanthopoma brachysoma	Gadus ogac	Parataeniophorus gulosus	Beryx decadactylus	(12)(34)	[20]	•	(14)(23)	•	0.03032
15	Branchiostegus albus	Coreoperca kawamebari	Fundulus diaphanus	Indostomus paradoxus	(12)(34)	[20]	•	(14)(23)	•	0.02996
16	Ceratias uranoscopus	Elassoma evergladei	Salangichthys microdon	Pantodon buchholzi	(12)(34)	[20]	•	(13)(24)	•	0.02971
17	Gila conspersa	Barbus barbus	Psilorhynchus homaloptera	Alepocephalus bairdii	(12)(34)	[23]	•	(13)(24)	•	0.02965
18	Nessorhamphus ingolfianus	Albula glossodonta	Sprattus sprattus	Galaxias maculatus	(12)(34)	[20]	•	(13)(24)	•	0.02928
19	Oryzias sinensis	Diaphus splendidus	Spratelloides delicatulus	Cyema atrum	(12)(34)	[20]	•	(14)(23)	•	0.02904
20	Oncorhynchus clarkii henshawi	Diplophos taenia	Papyrocranus congoensis	Chirocentrus dorab	(12)(34)	[20]	•	(13)(24)	•	0.02820
21	Clinocottus analis	Rudarius ercodes	Barbus trimaculatus	Chirocentrus dorab	(12)(34)	[20]	•	(14)(23)	•	0.05785
22	Fundulus diaphanus	Lagocephalus wheeleri	Eutaeniophorus sp. 033-Miya	Alepocephalus bicolor	(12)(34)	[20]	•	(14)(23)	•	0.02784
23	Oryzias sinensis	Zu cristatus	Alepocephalus bairdii	Albula glossodonta	(12)(34)	[20]	•	(13)(24)	•	0.02770

TABLE II: Same as Table I, but for actinopterygii (ray-finned fishes).

hexapoda (insects etc.)										
	species names						classification predicted by			
Nr.	species 1	species 2	species 3	species 4	“true”	Ref.	$d^{(\log-\mathrm{MI})}$	$d^{(\log-\det)}$	$d^{(\mathrm{NSD})}$	$S_{(\log-\det,\log-\mathrm{MI})}$
1	Reticulitermes santonensis	Periplaneta fuliginosa	Ostrinia nubilalis	Japyx solifugus	(12)(34)	[24, 25]	•	(14)(23)	•	0.04653
2	Reticulitermes flavipes	Periplaneta fuliginosa	Anoplophora glabripennis	Japyx solifugus	(12)(34)	[24, 25]	•	(14)(23)	•	0.04556
3	Reticulitermes santonensis	Periplaneta fuliginosa	Trachypachus holmbergi	Japyx solifugus	(12)(34)	[24, 25]	•	(14)(23)	•	0.04494
4	Reticulitermes virginicus	Periplaneta fuliginosa	Simosyrphus grandicornis	Japyx solifugus	(12)(34)	[24, 25]	•	(14)(23)	•	0.04478
5	Reticulitermes hageni	Periplaneta fuliginosa	Simosyrphus grandicornis	Japyx solifugus	(12)(34)	[24, 25]	•	(14)(23)	•	0.04414
6	Reticulitermes santonensis	Periplaneta fuliginosa	Drosophila sechellia	Japyx solifugus	(12)(34)	[24, 25]	•	(14)(23)	(14)(23)	0.04400
7	Reticulitermes virginicus	Periplaneta fuliginosa	Ostrinia nubilalis	Japyx solifugus	(12)(34)	[24, 25]	•	(14)(23)	•	0.04252
8	Rhopaea magnicornis	Pyrophorus divergens	Coreana raphaelis	Reticulitermes flavipes	(12)(34)	[24, 25]	•	(13)(24)	•	0.03622
9	Pyrophorus divergens	Anoplophora glabripennis	Drosophila sechellia	Reticulitermes hageni	(12)(34)	[24, 25]	•	(14)(23)	(14)(23)	0.03622
10	Reticulitermes santonensis	Periplaneta fuliginosa	Gampsocleis gratiosa	Ostrinia furnacalis	(12)(34)	[24, 25]	•	(13)(24)	•	0.03444
11	Bactrocera oleae	Ceratitis capitata	Drosophila mauritiana	Reticulitermes hageni	(12)(34)	[26]	•	(14)(23)	•	0.03332
12	Reticulitermes santonensis	Simosyrphus grandicornis	Campodea fragilis	Gomphiocephalus hodgsoni	(12)(34)	[24, 25]	•	(13)(24)	•	0.03269
13	Rhopaea magnicornis	Simosyrphus grandicornis	Reticulitermes hageni	Pachypsylla venusta	(12)(34)	[24, 25]	(14)(23)	•	•	0.03258
14	Rhopaea magnicornis	Pyrophorus divergens	Reticulitermes hageni	Simosyrphus grandicornis	(12)(34)	[24, 25]	•	(14)(23)	•	0.03166
15	Gastrimargus marmoratus	Gryllotalpa orientalis	Polystoechotes punctatus	Siphlonurus immanis	(12)(34)	[25, 27]	(13)(24)	•	(13)(24)	0.03141
16	Neuroctenus parus	Drosophila sechellia	Atelura formicaria	Orchesella villosa	(12)(34)	[25, 27]	(13)(24)	(14)(23)	(14)(23)	0.02949
17	Siphlonurus immanis	Davidius lunatus	Reticulitermes flavipes	Ceratitis capitata	(12)(34)	[25, 27]	•	(14)(23)	(14)(23)	0.02785
18	Tetraphalerus bruchi	Sphaerius sp. BT0074	Atelura formicaria	Onychiurus orientalis	(12)(34)	[25, 27]	•	(13)(24)	•	0.02545

TABLE III: Same as Table I, but for hexapoda (insects etc.).

sauropsida (reptiles, birds)										
	species names						classification predicted by			
Nr.	species 1	species 2	species 3	species 4	“true”	Ref.	$d^{(\log-\mathrm{MI})}$	$d^{(\log-\det)}$	$d^{(\mathrm{NSD})}$	$S_{(\log-\det,\log-\mathrm{MI})}$
1	Trionyx triunguis	Dogania subplana	Lissemys punctata	Polychrus marmoratus	(12)(34)	[28]	•	(13)(24)	•	0.03026
2	Pelodiscus sinensis	Dogania subplana	Lissemys punctata	Indotestudo elongata	(12)(34)	[28]	•	(14)(23)	•	0.02877
3	Pelodiscus sinensis	Dogania subplana	Lissemys punctata	Chinemys reevesi	(12)(34)	[28]	•	(14)(23)	•	0.02874
4	Pelodiscus sinensis	Dogania subplana	Lissemys punctata	Pyxidea mouhotii	(12)(34)	[28]	•	(14)(23)	•	0.02765
5	Trionyx triunguis	Dogania subplana	Lissemys punctata	Cygnus atratus	(12)(34)	[28]	•	(14)(23)	•	0.02752
6	Pterodroma brevirostris	Ciconia ciconia	Tachybaptus novaehollandiae	Anas platyrhynchos	(12)(34)	[29]	•	(13)(24)	•	0.02706
7	Pterodroma brevirostris	Eudyptula minor	Pteroglossus azara flavirostris	Apteryx haastii	(12)(34)	[29]	•	(14)(23)	•	0.02666
8	Lissemys punctata	Dogania subplana	Indotestudo forstenii	Gallus sonneratii	(12)(34)	[25, 27]	(14)(23)	•	(14)(23)	0.02641
9	Tragopan caboti	Syrmaticus ellioti	Gallus varius	Manouria emys	(12)(34)	[30]	•	(13)(24)	•	0.02623
10	Eudyptula minor	Ciconia ciconia	Pteroglossus azara flavirostris	Dromaius novaehollandiae	(12)(34)	[29]	(14)(23)	•	(14)(23)	0.02573
11	Pelodiscus sinensis	Dogania subplana	Lissemys punctata	Cyclemys atripons	(12)(34)	[28]	•	(14)(23)	•	0.02568
12	Tragopan caboti	Syrmaticus humiae	Manouria emys	Dogania subplana	(12)(34)	[25, 27]	•	(14)(23)	•	0.02435
13	Pterodroma brevirostris	Eudyptula minor	Pteroglossus azara flavirostris	Acrocephalus scirpaceus	(12)(34)	[29]	•	(13)(24)	•	0.02413
14	Cathartes aura	Ciconia ciconia	Tachybaptus novaehollandiae	Cairina moschata	(12)(34)	[25, 27]	•	(14)(23)	•	0.02391
15	Pteroglossus azara flavirostris	Dryocopus pileatus	Eudyptula minor	Indotestudo elongata	(12)(34)	[25, 27]	(14)(23)	•	(14)(23)	0.02378
16	Tragopan caboti	Syrmaticus humiae	Gallus gallus gallus	Leiocephalus personatus	(12)(34)	[30]	•	(13)(24)	•	0.02344
17	Phoenicopterus ruber roseus	Cathartes aura	Trogon viridis	Anseranas semipalmata	(12)(34)	[27, 29]	•	(14)(23)	•	0.02304
18	Cuora flavomarginata	Cuora aurocapitata	Pyxidea mouhotii	Syrmaticus humiae	(12)(34)	[25, 27]	(13)(24)	•	(13)(24)	0.01933

TABLE IV: Same as Table I, but for sauropsida (reptiles, birds).

amphibia										
	species names						classification predicted by			
Nr.	species 1	species 2	species 3	species 4	“true”	Ref.	$d^{(\log-\mathrm{MI})}$	$d^{(\log-\det)}$	$d^{(\mathrm{NSD})}$	$S_{(\log-\det,\log-\mathrm{M}}$[illegible]
1	Hydromantes brunus	Oedipina poelzi	Batrachuperus yenyuanensis	Xenopus (sil.) tropicalis	(12)(34)	[31]	•	(13)(24)	•	0.02343
2	Hydromantes brunus	Paramesotriton hongkongensis	Batrachuperus yenyuanensis	Alytes obstetricans pertinax	(12)(34)	[31]	(14)(23)	(13)(24)	(14)(23)	0.02340
3	Plethodon cinereus	Desmognathus fuscus	Ranodon sibiricus	Typhlonectes natans	(12)(34)	[31]	•	(14)(23)	•	0.02308
4	Eurycea bislineata	Gyrinophilus porphyriticus	Desmognathus fuscus	Xenopus (sil.) tropicalis	(12)(34)	[31]	•	(13)(24)	•	0.02265
5	Hydromantes brunus	Oedipina poelzi	Pachyhynobius shangchengensis	Bufo gargarizans	(12)(34)	[31]	•	(13)(24)	(14)(23)	0.02196
6	Ranodon sibiricus	Xenopus (sil.) tropicalis	Scolecomorphus vittatus	Typhlonectes natans	(12)(34)	[31]	•	(13)(24)	(14)(23)	0.02093
7	Bufo gargarizans	Pachyhynobius shangchengensis	Scolecomorphus vittatus	Typhlonectes natans	(12)(34)	[31]	•	(14)(23)	(13)(24)	0.02080
8	Plethodon cinereus	Desmognathus wrighti	Ichthyophis glutinosus	Typhlonectes natans	(12)(34)	[31]	•	(13)(24)	•	0.01940
9	Ranodon sibiricus	Xenopus (sil.) tropicalis	Scolecomorphus vittatus	Rhinatrema bivittatum	(12)(34)	[31]	•	(14)(23)	(13)(24)	0.01889
10	Oedipina poelzi	Thorius n. sp. RLM-2004	Pseudotriton ruber	Gegeneophis ramaswamii	(12)(34)	[32]	(14)(23)	•	(14)(23)	0.01684

TABLE V: Same as Table I, but for amphibia.

nematoda										
	species names						classification predicted by			
Nr.	species 1	species 2	species 3	species 4	"true"	Ref.	$d^{(\log-\mathrm{MI})}$	$d^{(\log-\det)}$	$d^{(\mathrm{NSD})}$	$S_{(\log-\det,\log-\mathrm{MI})}$
1	Metastrongylus salmi	Trichostrongylus vitrinus	Toxocara malaysiensis	Caenorhabditis elegans	(12)(34)	[25, 27]	•	(13)(24)	•	0.05208
2	Metastrongylus salmi	Trichostrongylus vitrinus	Toxocara canis	Caenorhabditis elegans	(12)(34)	[25, 27]	•	(13)(24)	•	0.04941
3	Metastrongylus salmi	Mecistocirrus digitatus	Toxocara malaysiensis	Caenorhabditis elegans	(12)(34)	[25, 27]	•	(13)(24)	•	0.04938
4	Steinernema carpocapsae	Angiostrongylus cantonensis	Ascaris suum	Caenorhabditis elegans	?	–	(13)(24)	(14)(23)	(13)(24)	0.04833
5	Mecistocirrus digitatus	Metastrongylus salmi	Toxocara cati	Caenorhabditis elegans	(12)(34)	[25, 27]	•	(14)(23)	•	0.04296
6	Metastrongylus salmi	Cylicocyclus insignis	Toxocara canis	Caenorhabditis elegans	(12)(34)	[25, 27]	•	(13)(24)	•	0.04269
7	Metastrongylus salmi	Ancylostoma caninum	Toxocara canis	Caenorhabditis elegans	(12)(34)	[25, 27]	•	(13)(24)	•	0.04269
8	Trichostrongylus vitrinus	Angiostrongylus cantonensis	Toxocara canis	Caenorhabditis elegans	(12)(34)	[25, 27]	•	(14)(23)	•	0.04196
9	Teladorsagia circumcincta	Angiostrongylus costaricensis	Toxocara canis	Caenorhabditis elegans	(12)(34)	[25, 27]	•	(14)(23)	•	0.03318
10	Metastrongylus salmi	Necator americanus	Toxocara cati	Caenorhabditis elegans	(12)(34)	[25, 27]	•	(13)(24)	•	0.03301
11	Teladorsagia circumcincta	Metastrongylus salmi	Toxocara cati	Caenorhabditis elegans	(12)(34)	[25, 27]	•	(14)(23)	•	0.03248
12	Chabertia ovina	Strongylus vulgaris	Cooperia oncophora	Angiostrongylus cantonensis	(12)(34)	[27, 33]	•	(13)(24)	•	0.03171
13	Strongylus vulgaris	Oesophagostomum dentatum	Ascaris suum	Trichostrongylus vitrinus	(12)(34)	[33]	•	(13)(24)	•	0.03113

TABLE VI: Same as Table I, but for nematoda.

crustacea										
	species names						classification predicted by			
Nr.	species 1	species 2	species 3	species 4	“true”	Ref.	$d^{(\log-\mathrm{MI})}$	$d^{(\log-\det)}$	$d^{(\mathrm{NSD})}$	$S_{(\log-\det,\log-\mathrm{MI})}$
1	Harpiosquilla harpax	Squilla mantis	Halocaridina rubra	Callinectes sapidus	(12)(34)	[25, 27]	(14)(23)	•	(14)(23)	0.01691
2	Macrobrachium rosenbergii	Scylla tranquebarica	Marsupenaeus japonicus	Marsupenaeus japonicus	(12)(34)	[25, 27]	•	(14)(23)	•	0.01483
3	Exopalaemon carinicauda	Scylla tranquebarica	Gonodactylus chiragra	Litopenaeus vannamei	(12)(34)	[25, 27]	•	(13)(24)	•	0.01466
4	Callinectes sapidus	Charybdis japonica	Portunus trituberculatus	Pseudocarcinus gigas	(12)(34)	[34]	•	(13)(24)	•	0.01447
5	Litopenaeus vannamei	Charybdis japonica	Squilla mantis	Daphnia pulex	(12)(34)	[25, 27]	(13)(24)	•	(13)(24)	0.01435
6	Exopalaemon carinicauda	Portunus trituberculatus	Fenneropenaeus chinensis	Lysiosquillina maculata	(12)(34)	[25, 27]	(13)(24)	•	(13)(24)	0.02397
7	Portunus trituberculatus	Litopenaeus vannamei	Squilla mantis	Daphnia pulex	(12)(34)	[25, 27]	(13)(24)	•	(13)(24)	0.01356

TABLE VII: Same as Table I, but for crustacea.

mollusca										
	species names						classification predicted by			
Nr.	species 1	species 2	species 3	species 4	“true”	Ref.	$d^{(\log-\mathrm{MI})}$	$d^{(\log-\det)}$	$d^{(\mathrm{NSD})}$	$S_{(\log-\det,\log-\mathrm{MI})}$
1	Lophiotoma cerithiformis	Terebra dimidiata	Nassarius reticulatus	Conus textile	(12)(34)	[35]	•	(13)(24)	•	0.02338
2	Ascobulla fragilis	Elysia chlorotica	Biomphalaria tenagophila	Onchidella celtica	(12)(34)	[27, 36]	•	(13)(24)	•	0.02090
3	Biomphalaria tenagophila	Siphonaria pectinata	Ascobulla fragilis	Terebra dimidiata	?	[27, 36]	(14)(23)	(13)(24)	(14)(23)	0.01933
4	Lophiotoma cerithiformis	Terebra dimidiata	Nassarius reticulatus	Conus borgesi	(12)(34)	[35]	•	(13)(24)	•	0.01892
5	Fusiturris similis	Terebra dimidiata	Cymatium parthenopeum	Conus textile	(12)(34)	[35]	•	(14)(23)	•	0.01817
6	Ilyanassa obsoleta	Terebra dimidiata	Cancellaria cancellata	Oncomelania hupensis	(12)(34)	[37]	•	(14)(23)	•	0.01773
7	Terebra dimidiata	Rapana venosa	Oncomelania hup. robertsoni	Cancellaria cancellata	(12)(34)	[37]	•	(14)(23)	•	0.01656
8	Ascobulla fragilis	Aplysia californica	Biomphalaria tenagophila	Pupa strigosa	(12)(34)	[37]	•	(13)(24)	•	0.01616

TABLE VIII: Same as Table I, but for mollusca.

platyhelminthes										
	species names						classification predicted by			
Nr.	species 1	species 2	species 3	species 4	“true”	Ref.	$d^{(\log-\mathrm{MI})}$	$d^{(\log-\det)}$	$d^{(\mathrm{NSD})}$	$S_{(\log-\det,\log-\mathrm{MI})}$
1	Taenia pisiformis	Taenia crassiceps	Echinococcus oligarthrus	Hymenolepsis diminuta	(12)(34)	[38]	(14)(23)	•	(14)(23)	0.02330
2	Taenia pisiformis	Taenia asiatica	Echinococcus canadensis	Taenia crassiceps	(12)(34)	[38, 39]	(13)(24)	(14)(23)	(13)(24)	0.01957
3	Taenia pisiformis	Taenia crassiceps	Echinococcus multilocularis	Hymenolepsis diminuta	(12)(34)	[38]	(14)(23)	•	(14)(23)	0.01930
4	Taenia pisiformis	Taenia crassiceps	Spirometra erinaceieuropaei	Echinococcus vogeli	(12)(34)	[38]	(13)(24)	•	•	0.01831
5	Taenia pisiformis	Taenia asiatica	Taenia crassiceps	Echinococcus vogeli	(12)(34)	[38, 39]	•	(13)(24)	•	0.01734
6	Taenia pisiformis	Taenia multiceps	Taenia hydatagenia	Echinococcus shiquicus	(12)(34)	[39]	(14)(23)	•	(14)(23)	0.01625
7	Taenia pisiformis	Taenia saginata	Echinococcus multilocularis	Diphyllobothrium latum	(12)(34)	[38]	•	(14)(23)	•	0.01174
8	Echinococcus multilocularis	Echinococcus granulosus	Echinococcus oligarthrus	Taenia asiatica	(12)(34)	[40]	•	(14)(23)	•	0.00998

TABLE IX: Same as Table I, but for platyhelminthes.

	U. maritimus	U. americanus	U. thibetanus	O. cuniculus
	$d^{(NSD)}$			
U. maritimus	–	0.23364	0.21966	0.59586
U. americanus	0.23444	–	0.19891	0.57536
U. thibetanus	0.21851	0.19539	–	0.58062
O. cuniculus	0.59586	0.57536	0.58062	–
	$d^{(\log-\mathrm{MI})}$			
U. maritimus	–	0.26611	0.24802	0.90600
U. americanus	0.26611	–	0.22178	0.85651
U. thibetanus	0.24802	0.22178	–	0.86897
O. cuniculus	0.90600	0.86897	0.86897	–

TABLE X: Two distance matrices for the same quartet obtained with the normalized Shannon distance (top) and with its log-transformed version (bottom). With the first, one obtains the wrong classification ((*americanus, thibetanus*), *maritimus*) due to the Felsenstein phenomenon, since $d^{(\mathrm{NSD})}$ is not additive. With the second, additivity is approximately restored, but noise is amplified, resulting in the wrong classification ((*maritimus, thibetanus*), *americanus*). The correct classification would be [41] ((*maritimus, americanus*), *thibetanus*).
